# Supplementary material for: Socioeconomic and ethnic differences in children’s vigorous intensity physical activity: a cross-sectional analysis of the UK Millennium Cohort Study
Source: BMJ Open. 2019 May 27;9(5):e027627. doi: 10.1136/bmjopen-2018-027627 (PMC6549689; doi:10.1136/bmjopen-2018-027627)
Supplement: Supplementary file 1 [file bmjopen-2018-027627supp001.pdf]

Supplementary Files: Socio-economic and ethnic differences in children's vigorous intensity physical activity: a cross-sectional analysis of the UK Millennium Cohort Study

Contents:

Supplementary File 1: Full multivariable linear regression models for mean minutes of VPA and MPA overall, on weekdays and on weekend days, by a) ethnic subgroups b) maternal education and c) household equivalised income (n=5172) within the Millennium Cohort Accelerometer Study, 4th follow-up (2008-09)

Supplementary File 2: Multivariable linear regression models for mean minutes of VPA with additional adjustments for BMI z-score overall, on weekdays and on weekend days, by ethnic subgroups maternal education and household equivalised income (n=5149) within the Millennium Cohort Accelerometer Study, 4th follow-up (2008-09)

Supplementary File 3: Multivariable linear regression models for BMI z-score by minutes of daily VPA and MPA (n=5149) within the Millennium Cohort Accelerometer Study, 4th follow-up (2008-09)

Supplementary File 1a) Multivariable linear regression models for mean minutes of VPA and MPA overall, weekdays and weekend days, by ethnic subgroups (n=5172) within the Millennium Cohort Accelerometer Study, 4th follow-up (2008-09)

**By ethnic group**			note: .01 - ***; .05 - **; .1 - *;		
**VPA (mins/day)**			**MPA (mins/day)**		
**All valid days**					
**ref cat: White**	**coef**	**se**	**ref cat: White**	**coef**	**se**
Mixed	1.468*	0.782	Mixed	-2.223**	1.036
Indian	0.738	0.984	Indian	-3.938***	1.168
Pakistani & Bangladeshi	-3.344***	0.673	Pakistani & Bangladeshi	2.089**	0.900
Black or Black British	-1.675	1.168	Black or Black British	3.009***	0.988
Other ethnic group	-2.267**	0.966	Other ethnic group	1.087	1.728
MPA (mins/day)	0.584***	0.018	VPA (mins/day)	0.814***	0.020
Weartime (mins/day)	0.000	0.003	Weartime (mins/day)	0.014***	0.003
Age (yrs)	1.079**	0.535	Age (yrs)	-1.876***	0.657
Sex of participant	0.700**	0.292	Sex of participant	-5.064***	0.320
Season of measurement	0.042	0.165	Season of measurement	-1.727***	0.177
_cons	-14.150***	4.293	_cons	41.945***	5.295
r2	0.515		r2	0.565	
Number of observations	5,172		Number of observations	5,172	
**Weekdays**					
**ref cat: White**	**coef**	**se**	**ref cat: White**	**coef**	**se**
Mixed	1.569*	0.871	Mixed	-2.001*	1.138
Indian	0.578	0.923	Indian	-3.463***	1.106
Pakistani & Bangladeshi	-3.451***	0.743	Pakistani & Bangladeshi	1.998**	0.965
Black or Black British	-2.076*	1.223	Black or Black British	3.098***	1.167
Other ethnic group	-3.069***	1.145	Other ethnic group	1.888	1.891
Weekday MPA (mins/day)	0.584***	0.022	Weekday VPA (mins/day)	0.789***	0.021
Weekday weartime (mins/day)	-0.000	0.003	Weekday weartime (mins/day)	0.015***	0.003
Age (yrs)	0.951*	0.576	Age (yrs)	-1.331*	0.703
Sex of participant	0.739**	0.311	Sex of participant	-5.066***	0.332
Season of measurement	0.026	0.171	Season of measurement	-1.693***	0.184
_cons	-12.724***	4.553	_cons	37.062***	5.484
r2	0.497		r2	0.543	
Number of observations	5,172		Number of observations	5,172	
**Weekend days**					
**ref cat: White**	**coef**	**se**	**ref cat: White**	**coef**	**se**
Mixed	0.434	0.944	Mixed	-2.131	1.484
Indian	0.757	1.499	Indian	-4.463**	1.785
Pakistani & Bangladeshi	-3.001***	0.692	Pakistani & Bangladeshi	2.024*	1.198
Black or Black British	-0.035	1.736	Black or Black British	1.766	1.390
Other ethnic group	-0.141	1.091	Other ethnic group	-1.339	2.059
Weekend MPA (mins/day)	0.540***	0.016	Weekend VPA (mins/day)	0.956***	0.023
Weekend weartime (mins/day)	-0.002	0.002	Weekend weartime (mins/day)	0.017***	0.003
Age (yrs)	1.389**	0.689	Age (yrs)	-3.486***	0.918
Sex of participant	0.103	0.331	Sex of participant	-4.643***	0.466
Season of measurement	-0.205	0.217	Season of measurement	-1.483***	0.270
_cons	-12.259**	5.199	_cons	48.524***	7.067
r2	0.546		r2	0.572	
Number of observations	5,172		Number of observations	5,172	

Supplementary File 1b) Multivariable linear regression models for mean minutes of VPA and MPA overall, weekdays and weekend days, by level of maternal education (n=5172) within the Millennium Cohort Accelerometer Study, 4th follow-up (2008-09)

**By level of maternal education**			note: .01 - ***; .05 - **; .1 - *;		
**VPA (mins/day)**			**MPA (mins/day)**		
**All valid days**					
**ref cat: No qualifications**	**coef**	**se**	**ref cat: No qualifications**	**coef**	**se**
Low	1.314**	0.559	Low	-0.950	0.836
Medium	1.653***	0.572	Medium	-1.483*	0.851
High	1.812***	0.532	High	-1.689**	0.815
Higher	2.957***	0.769	Higher	-2.738***	0.925
Overseas qual.	2.276*	1.314	Overseas qual.	-1.957	1.334
None of these	-0.448	0.674	None of these	1.978*	1.035
MPA (mins/day)	0.587***	0.018	VPA (mins/day)	0.815***	0.019
Weartime (mins/day)	-0.002	0.003	Weartime (mins/day)	0.014***	0.003
Age (yrs)	1.044*	0.543	Age (yrs)	-1.901***	0.656
Sex of participant	0.681**	0.297	Sex of participant	-5.043***	0.325
Season of measurement	0.081	0.164	Season of measurement	-1.736***	0.179
_cons	-14.404***	4.418	_cons	42.866***	5.471
r2	0.515		r2	0.567	
Number of observations	5,172		Number of observations	5,172	
**Weekdays**					
**ref cat: No qualifications**	**coef**	**se**	**ref cat: No qualifications**	**coef**	**se**
Low	1.314**	0.616	Low	-0.944	0.893
Medium	1.717***	0.628	Medium	-1.384	0.907
High	1.880***	0.585	High	-1.732**	0.869
Higher	3.045***	0.843	Higher	-2.708***	0.962
Overseas qual.	2.123	1.314	Overseas qual.	-2.293	1.407
None of these	-0.597	0.727	None of these	1.878*	1.116
Weekday MPA (mins/day)	0.588***	0.021	Weekday VPA (mins/day)	0.790***	0.021
Weekday weartime (mins/day)	-0.002	0.003	Weekday weartime (mins/day)	0.015***	0.003
Age (yrs)	0.910	0.584	Age (yrs)	-1.343*	0.697
Sex of participant	0.713**	0.316	Sex of participant	-5.038***	0.336
Season of measurement	0.067	0.170	Season of measurement	-1.703***	0.185
_cons	-12.864***	4.666	_cons	37.919***	5.647
r2	0.496		r2	0.545	
Number of observations	5,172		Number of observations	5,172	
**Weekend days**					
**ref cat: No qualifications**	**coef**	**se**	**ref cat: No qualifications**	**coef**	**se**
Low	1.335*	0.711	Low	-1.317	1.055
Medium	1.366*	0.736	Medium	-1.894*	1.112
High	1.627**	0.668	High	-1.711*	1.016
Higher	2.796***	0.884	Higher	-3.556***	1.194
Overseas qual.	2.694	1.714	Overseas qual.	-1.735	1.953
None of these	-0.065	0.868	None of these	2.348*	1.354
Weekend MPA (mins/day)	0.543***	0.016	Weekend VPA (mins/day)	0.955***	0.023
Weekend weartime (mins/day)	-0.002	0.002	Weekend weartime (mins/day)	0.017***	0.003
Age (yrs)	1.376**	0.698	Age (yrs)	-3.554***	0.927
Sex of participant	0.105	0.333	Sex of participant	-4.654***	0.467
Season of measurement	-0.171	0.219	Season of measurement	-1.495***	0.271
_cons	-13.489**	5.374	_cons	50.213***	7.259
r2	0.546		r2	0.575	
Number of observations	5,172		Number of observations	5,172	

Supplementary File 1c) Multivariable linear regression models for mean minutes of VPA and MPA overall, weekdays and weekend days, by annual household equivalised income (n=5172) within the Millennium Cohort Accelerometer Study, 4th follow-up (2008-09)

**By household equivalised income**			note: .01 - ***; .05 - **; .1 - *;		
**VPA (mins/day)**			**MPA (mins/day)**		
**All valid days**					
	**coef**	**se**		**coef**	**se**
Annual income/10000	0.579***	0.131	Annual income/10000	-0.976***	0.141
MPA (mins/day)	0.587***	0.018	VPA (mins/day)	0.812***	0.019
Weartime (mins/day)	-0.001	0.003	Weartime (mins/day)	0.014***	0.003
Age (yrs)	0.998*	0.540	Age (yrs)	-1.828***	0.654
Sex of participant	0.719**	0.299	Sex of participant	-5.109***	0.325
Season of measurement	0.103	0.166	Season of measurement	-1.772***	0.178
_cons	-14.095***	4.396	_cons	43.738***	5.339
r2	0.513		r2	0.566	
Number of observations	5,172		Number of observations	5,172	
**Weekdays**					
	**coef**	**se**		**coef**	**se**
Annual income/10000	0.613***	0.136	Annual income/10000	-0.990***	0.145
Weekday MPA (mins/day)	0.588***	0.022	Weekday VPA (mins/day)	0.786***	0.021
Weekday weartime (mins/day)	-0.002	0.003	Weekday weartime (mins/day)	0.015***	0.003
Age (yrs)	0.857	0.581	Age (yrs)	-1.272*	0.696
Sex of participant	0.753**	0.320	Sex of participant	-5.107***	0.336
Season of measurement	0.092	0.172	Season of measurement	-1.741***	0.184
_cons	-12.484***	4.629	_cons	38.716***	5.474
r2	0.494		r2	0.545	
Number of observations	5,172		Number of observations	5,172	
**Weekend days**					
	**coef**	**se**		**coef**	**se**
Annual income/10000	0.500***	0.162	Annual income/10000	-0.915***	0.210
Weekend MPA (mins/day)	0.542***	0.016	Weekend VPA (mins/day)	0.957***	0.023
Weekend weartime (mins/day)	-0.002	0.002	Weekend weartime (mins/day)	0.016***	0.003
Age (yrs)	1.346*	0.695	Age (yrs)	-3.477***	0.922
Sex of participant	0.140	0.334	Sex of participant	-4.701***	0.469
Season of measurement	-0.153	0.220	Season of measurement	-1.520***	0.272
_cons	-13.159**	5.327	_cons	50.629***	7.161
r2	0.545		r2	0.572	
Number of observations	5,172		Number of observations	5,172	

Supplementary file 2

Multivariable linear regression models for mean minutes of VPA with additional adjustments for BMI z-score overall, on weekdays and on weekend days, by ethnic subgroups maternal education and household equivalised income (n=5149) within the Millennium Cohort Accelerometer Study, 4th follow-up (2008-09)

Ethnicity						
	All valid days		Weekdays		Weekends	
	coef	se	coef	se	coef	se
Mixed	1.400*	0.754	1.503*	0.831	0.364	0.961
Indian	0.484	0.989	0.330	0.932	0.522	1.512
Pakistani & Bangladeshi	-3.616***	0.626	-3.735***	0.693	-3.240***	0.668
Black or Black British	-0.769	1.072	-1.124	1.139	0.678	1.680
Other ethnic group	-2.668***	0.981	-3.508***	1.161	-0.421	1.097
MPA (mins/day)	0.579***	0.018	0.581***	0.021	0.536***	0.015
Weartime (mins/day)	-0.000	0.003	-0.000	0.003	-0.002	0.002
Age (yrs)	1.069**	0.530	0.947*	0.571	1.374**	0.686
Sex of participant	0.686**	0.290	0.733**	0.309	0.099	0.330
Season of measurement	-0.007	0.162	-0.006	0.169	-0.295	0.212
BMI zscore	-0.882***	0.126	-0.932***	0.129	-0.702***	0.167
_cons	-13.169***	4.221	-11.801***	4.495	-11.436**	5.144
r2	0.523		1		1	
Number of observations	5,149		5,149		5,149	
**Maternal education**						
	All valid days		Weekdays		Weekends	
	coef	se	coef	se	coef	se
Low	1.385**	0.558	1.413**	0.616	1.332*	0.706
Medium	1.806***	0.572	1.882***	0.629	1.508**	0.732
High	1.841***	0.532	1.929***	0.586	1.612**	0.666
Higher	2.990***	0.765	3.093***	0.839	2.815***	0.880
Overseas qual.	2.278*	1.367	2.139	1.373	2.675	1.747
None of these	-0.452	0.668	-0.587	0.720	-0.101	0.863
MPA (mins/day)	0.584***	0.018	0.586***	0.021	0.540***	0.016
Weartime (mins/day)	-0.002	0.003	-0.002	0.003	-0.002	0.002
Age (yrs)	1.037*	0.536	0.908	0.577	1.363*	0.695
Sex of participant	0.669**	0.294	0.707**	0.313	0.102	0.332
Season of measurement	0.032	0.161	0.035	0.168	-0.260	0.213
BMI zscore	-0.876***	0.136	-0.933***	0.140	-0.668***	0.171
_cons	-13.655***	4.342	-12.143***	4.602	-12.804**	5.323
r2	0.523		0.504		0.549	
Number of observations	5,149		5,149		5,149	
**Household equivalised income**						
	All valid days		Weekdays		Weekends	
	coef	se	coef	se	coef	se
Annual income/10000	0.579***	0.131	0.613***	0.136	0.500***	0.162
MPA (mins/day)	0.587***	0.018	0.588***	0.022	0.542***	0.016
Weartime (mins/day)	-0.001	0.003	-0.002	0.003	-0.002	0.002
Age (yrs)	0.998*	0.540	0.857	0.581	1.346*	0.695
Sex of participant	0.719**	0.299	0.753**	0.320	0.140	0.334
Season of measurement	0.103	0.166	0.092	0.172	-0.153	0.220
_cons	-14.095***	4.396	-12.484***	4.629	-13.159**	5.327
R2	0.513		0.494		0.545	
Number of observations	5,172		5,172		5,172	

Supplementary file 3

Multivariable linear regression models for BMI z-score by minutes of daily VPA and MPA (n=5149) within the Millennium Cohort Accelerometer Study, 4th follow-up (2008-09), adjusted for age, sex and wear time

BMI z-score	β-coeff	[95% Conf	Interval]
Mean Minutes MPA/day	-0.002	-0.006	-0.001
Mean Minutes VPA/day	-0.012	-0.017	-0.007
