# Supplementary material for: Socioeconomic and ethnic differences in children’s vigorous intensity physical activity: a cross-sectional analysis of the UK Millennium Cohort Study
Source: BMJ Open. 2019 May 27;9(5):e027627. doi: 10.1136/bmjopen-2018-027627 (PMC6549689; doi:10.1136/bmjopen-2018-027627)
Supplement: Author's manuscript [file bmjopen-2018-027627.draft_revisions.pdf]

BMJ Open is committed to open peer review. As part of this commitment we make the peer review history of every article we publish publicly available.

When an article is published we post the peer reviewers' comments and the authors' responses online. We also post the versions of the paper that were used during peer review. These are the versions that the peer review comments apply to.

The versions of the paper that follow are the versions that were submitted during the peer review process. They are not the versions of record or the final published versions. They should not be cited or distributed as the published version of this manuscript.

BMJ Open is an open access journal and the full, final, typeset and author-corrected version of record of the manuscript is available on our site with no access controls, subscription charges or pay-per-view fees (http://bmjopen.bmj.com).

If you have any questions on BMJ Open's open peer review process please email info.bmjopen@bmj.com

BMJ Open

Socio-economic and ethnic differences in children's vigorous intensity physical activity: a cross-sectional analysis of the UK Millennium Cohort Study

Journal:	*BMJ Open*
Manuscript ID	bmjopen-2018-027627
Article Type:	Research
Date Submitted by the Author:	31-Oct-2018
Complete List of Authors:	Love, Rebecca; University of Cambridge Department of Public Health and Primary Care, UKCRC Centre for Diet and Activity Research (CEDAR), MRC Epidemiology Unit<br>Adams, J; University of Cambridge, Centre for Diet & Activity Research<br>Atkin, Andrew; University of East Anglia Faculty of Medicine and Health Sciences,<br>van Sluijs, Esther; University of Cambridge, MRC Epidemiology Unit
Keywords:	PUBLIC HEALTH, PREVENTIVE MEDICINE, Health policy < HEALTH SERVICES ADMINISTRATION & MANAGEMENT

SCHOLARONE™
Manuscripts

Title:

Socio-economic and ethnic differences in children's vigorous intensity physical activity: a cross-sectional analysis of the UK Millennium Cohort Study

Author names:

Rebecca Love (PhD Candidate), Jean Adams (Senior University Lecturer), Andrew J. Atkin (Lecturer in Behavioural Epidemiology), Esther MF van Sluijs (MRC Programme Leader)

Author affiliations:

MRC Epidemiology Unit and UKCRC Centre for Diet and Activity Research (CEDAR), University of Cambridge School of Clinical Medicine, Institute of Metabolic Science, Cambridge Biomedical Campus, Cambridge, UK

- (Rebecca Love, MSc; Jean Adams, PhD; Esther MF van Sluijs, PhD)

School of Health Sciences, Faculty of Medicine and Health Sciences, University of East Anglia, Norwich Research Park, Norwich NR4 7TJ, UK

- (Andrew J. Atkin, PhD)

Corresponding author:

Rebecca Love

MRC Epidemiology Unit & Centre for Diet and Activity Research (CEDAR)

University of Cambridge School of Clinical Medicine,

Box 285, Institute of Metabolic Science,

Cambridge Biomedical Campus,

Cambridge

CB2 0QQ

E-mail: rel54@medschl.cam.ac.uk

Telephone: 0122 3746877

Author email addresses:

Jean Adams: jma79@medschl.cam.ac.uk

Esther van Sluijs: esther.vansluijs@mrc-epid.cam.ac.uk

Andrew J Atkin: A.Atkin@uea.ac.uk

Word count:

For peer review only

2864

Competing interests:
All authors have no competing interests to declare.

Funding:
Funding for this study and the work of all authors was supported, wholly or in part, by the Centre for Diet and Activity Research (CEDAR), a UKCRC Public Health Research Centre of Excellence (087636/Z/08/Z; ES/G007462/1; MR/K023187/1). Funding from the British Heart Foundation, Department of Health, Economic and Social Research Council, Medical Research Council, and the Wellcome Trust, under the auspices of the UK Clinical Research Collaboration, is gratefully acknowledged. Rebecca Love is funded by a Gates Cambridge Scholarship. The work of Esther M F van Sluijs was supported by the Medical Research Council (MC_UU_12015/7). No funders were involved in the production of this manuscript.

For peer review only

ABSTRACT

Objective: To investigate socio-economic and ethnic differences in the intensity patterning of children's physical activity using objective accelerometer measurements.

Design: Nationally representative prospective cohort study.

Setting: UK children born between 2000 and 2002.

Participants: 5098 7-8-year-old children with valid accelerometer data for ≥10 h on ≥3 days, including one weekend day.

Main outcome measures: Time spent in vigorous physical activity (VPA) (>3841 counts per minute).

Explanatory measures: Maternal education, annual household OECD equivalised income, ethnicity.

Results: Multivariable linear regression models fitted to explore differences in average daily minutes of VPA (adjusted for moderate physical activity (MPA), mean accelerometer wear time, season of measurement, age and sex), revealed significantly higher amounts of VPA accumulated as a child's socioeconomic position increased (highest versus lowest level of maternal education: β: 2.84, p: 0.00; annual household equivalised income: β: 0.35, p: 0.00, per £10,000 annual increase). Additionally, children from certain minority ethnicities (Bangladeshi and Pakistani: β: -2.84, p: 0.00; black or black British: β:-1.49, p: 0.05) accrued less daily VPA compared with their white British counterparts.

Conclusions: The socioeconomic and ethnic patterning of vigorous activity observed in this study mirrors parallel inequalities in rates of childhood obesity. Given the stronger association of vigorous activity (VPA) with adiposity than of moderate activity (MPA), intensity specific differences may be contributing to widening inequalities in obesity. Accordingly, these findings suggest that the current global focus on overall MVPA may mask important behavioural inequalities.

For peer review only

STRENGTHS AND WEAKNESSES

- This is the first study to investigate socio-economic and ethnicity-related differences in children's vigorous intensity physical activity behaviour, accounting for moderate physical activity.

- We used data from the Millennium Cohort Study which used a stratified sampling design enabling adequate representation of socioeconomically disadvantaged and ethnic minority children.

- This is the largest available accelerometer dataset of objectively measured physical activity in UK children.

- Boys, certain ethnic minorities (Indian, Pakistan/Bangladeshi and Black Caribbean/African), and children living with only one parent were less likely to provide valid accelerometer data which may have affected the representativeness of our findings.

- The accelerometer measurements utilized are limited in their ability to classify behaviour, detect certain activities (e.g. cycling and swimming), upper body movements or changes in terrain.

For peer review only

BACKGROUND

Over the past four decades, global prevalence of childhood obesity has increased tenfold.[1] Predictions forecast that if current trends continue, by 2030, 60% of the global adult population will be overweight or obese.[2] Given that obesity in childhood is associated with premature mortality and physical morbidity in adulthood,[3] tackling childhood obesity is increasingly a public health priority for governments.[4]

Of increasing concern are widening inequalities in obesity prevalence. By age 11, UK children from lower income families are three times as likely to be obese than their more advantaged counterparts.[5] These differences progressively worsen with age, fuelling inequality in health across the life-course.[6,7] There are also stark ethnic and racial differences in childhood adiposity, with higher rates of obesity within certain ethnic minorities including Black British and Bangladeshi.[8,9] It is suggested that these differences are partially linked to existing disparities in socioeconomic resources between ethnic groups.[9] At present, there is limited understanding of the modifiable factors driving socio-economic and ethnicity-related disparities in childhood obesity.

Physical activity is a key behavioural driver of cardiovascular disease risk in children.[10] Evidence of an association with obesity is less clear.[11,12] However, evidence suggests that vigorous intensity activity (VPA) may be more strongly associated with reduced waist circumference and adiposity relative to lower intensity activity, including moderate intensity activity (MPA).[13–17]

Previous research using objectively measured physical activity has suggested no socioeconomic patterning in children's adherence to the international guidelines to engage in 'moderate-to-vigorous intensity activity (MVPA) for at least 60 minutes per day'.[18–20] In addition, time spent in MVPA only explains a small portion of the socioeconomic gradient in overweight risk present within UK children.[21] There is some evidence of ethnic differences in activity levels,[22] but these analyses are confined to the use of the aggregate measure of MVPA to quantify activity levels. However, moderate physical activity (MPA) and vigorous physical activity (VPA) are generally accumulated through different types of activities (e.g. walking to school vs. sport participation), which may be differently distributed in population subgroups. In high-income countries, children's participation in the organized activities that tend to drive VPA is socially patterned due to unequal access, support and costs.[23–25]

The importance of intensity specific differences of activity behaviour in explaining socioeconomic and ethnic inequalities in health remains underexplored. The need for understanding of these differences is strengthened by evidence suggesting that the age-related decreases in VPA observed annually into adulthood are more pronounced in non-white and socio-economically deprived children, than their counterparts.[26]

We propose that socio-economic and ethnic differences in children's activity intensity could help to explain known socio-economic and ethnic gradients in obesity. Thus, the objective of this

For peer review only - http://bmjopen.bmj.com/site/about/guidelines.xhtml

study was to investigate if daily VPA, adjusted for minutes of MPA performed, differs by socioeconomic position or ethnicity in a large sample of UK children aged 7 years.

For peer review only

METHODS

Sample

This analysis used data from the Millennium Cohort Study (MCS), a nationally representative, longitudinal study of children born in the United Kingdom between September 2000 and January 2002.[27] MCS was developed to track the social, economic and health experiences facing children born at the start of the 21st century. The original cohort included 18,818 children. Data was first collected when cohort participants were 9 months old through a home-based interview (72% of those initially contacted provided information). Subsequent follow-up interviews at ages 3, 5, 7 and 11 years were conducted in the home environment with the main respondent (primarily the natural mother, and where applicable the partner).[28]

To ensure adequate representation of all four UK countries, including disadvantaged and minority populations, a stratified clustered sampling design was used to oversample children living in Wales, Scotland and Northern Ireland, disadvantaged areas, and communities with high proportions of minority ethnic groups. The MCS received ethics approval from the South West and London Multi-centre Research Ethnics Committees, UK and the Yorkshire Research Ethics Committee. The analyses presented here used data from follow-up 4 at age 7 years (MCS4) and did not require additional ethical approval.

Physical Activity

At MCS4 participating children (n=13 681) were invited to participate in an accelerometer study. Consenting children were sent a pre-programmed Actigraph GT1M accelerometer (Actigraph, Pensacola, Florida), set to record data at 15-second epochs. These accelerometers have been shown to be reliable and valid in comparison to measures of physical activity derived from heart rate monitoring, indirect and room calorimetry, and doubly labelled water.[29–31] Children were instructed to wear the accelerometer on an elastic belt around their waist for 7 days throughout all waking hours (except during aquatic activities or contact sports), and to return it by post. Data was collected over a 15-month period between May 2008 – August 2009.

Data was downloaded using Actigraph software V.3.8.3 (Actigraph, Pensacola, Florida, USA) and processed according to predetermined criteria, using the R statistical package pawacc.[32] Non-wear time, defined as 20 minutes or more of consecutive zero activity counts, were excluded from the analysis. Counts were separated into sedentary (<100 counts per min (cpm)), moderate (2240-3840 cpm) and vigorous (>3841 cpm) categories.[32] To ensure reliable estimates of activity, the sample was restricted to singleton children with 3 valid days of data (≥ 10 h/day, including at least 1 weekend day).[33] Values above a threshold of ≥11715 cpm were excluded, as were children with ≥150 mean mins of MVPA per day.

For peer review only - http://bmjopen.bmj.com/site/about/guidelines.xhtml

Sociodemographic variables

Information pertaining to socioeconomic position and ethnicity were collected at MCS4. Socio-economic position was measured using maternal education and equivalised household income. Maternal education captures the socioeconomic circumstances that accrue to a child and is advantageous as it can be applied to mothers irrespective of whether or not they are in paid employment at the time of interview.[34] Maternal education was categorized into five groups: none (qualifications less than those currently expected when leaving school at 16 years); low (qualifications comparable to those currently expected when leaving school at 16 years); medium (qualifications comparable to those currently expected when leaving school at 18 years); high (qualifications greater than medium, but not higher); and higher (any higher educational qualifications).[35] Annual household income was equivalized for household composition based on guidance from the Organisation for Economic Co-Operation and Development (OECD).[27] Ethnicity was parent-reported and categorised: white, any mixed, Indian, Pakistani or Bangladeshi, black or black British, or other, in accordance with guidelines from the Office for National Statistics.[36]

Statistical analysis

Multivariable linear regression models were fitted to analyse differences in absolute minutes of VPA achieved across socio-economic and ethnic groups, adjusting for MPA, mean accelerometer wear time, season of measurement, age and sex. Separate models were run for each exposure variable (maternal education, equivalised household income, ethnicity). Models were also run separately for week and weekend days as there is evidence that children accumulate physical activity differently on weekdays and weekend days.[37] Further, it is possible that different socio-economic and ethnic subgroups of children engage in different types of activities with distinct weekly patterning (e.g. weekend sports). To investigate potential differences in effect by gender, interactions were run across all models. Additional adjustments for BMI and MVPA did not affect the results of the models. VPA was non-normally distributed and therefore all regression models were additionally run using a log-transformed VPA outcome variable. Results were essentially unchanged thus non-transformed analyses are presented here for ease of interpretation. All analyses were conducted using STATA 15.1 software, with survey commands used to account for the stratified clustered design of MCS and to obtain robust standard errors.

For peer review only

RESULTS

Of the 12,872 children that consented to the accelerometer study, 9,772 returned the accelerometers, with a final sample of 6,497 children following in-house processing by MCS.[38] Application of our study inclusion criteria resulted in an analytic sample of 5,098 children. This drop was predominately driven by our requirement participant's '3 or more valid days', include one weekend day to enable comparisons across weekend and weekdays. On average, children in the weighted sample were 6.8 years of age (SD: 0.4) and 50% female (See Table 1). Overall, 14.1% of girls and 11.7% of boys were overweight, whilst 3.7% and 2.9% were obese. These classifications were made through application of the International Obesity Taskforce age and gender specific BMI cut-points.[39] The sample included children from each country across the United Kingdom (England, Wales, Scotland and Northern Ireland). Socio-demographic and physical activity summary characteristics of the analytic sample are outlined in Table 1 and 2, respectively. These two tables of sample characteristics are based on the weighted sample given that all our analyses are weighted. Drop out analyses showed that those participants included in the analytic sample (n=5098) were more likely to come from a higher income household, have mothers with higher levels of education, and be male when compared to participants who provided accelerometer data but did not meet the criteria for the analytic sample (n=1577).

Multivariable linear regression models revealed significant differences in the minutes of VPA accumulated across socio-economic subgroups (Table 3). Significantly more minutes of VPA was accumulated in each level of maternal education compared to those whose mother indicated 'no qualifications'. This relationship was more pronounced on weekdays than on weekend days. Analyses by equivalized household income supported this, indicating significantly more time spent in VPA with increasing household income.

Pakistani & Bangladeshi children performed close to 3 minutes less daily VPA in comparison to white British children overall, on weekdays and weekend days. This difference was slightly more pronounced on weekdays, versus weekend days. Black and black British children also accumulated less daily VPA overall and on weekdays. In contrast, children from a mixed ethnic descent accumulated comparatively more minutes of VPA daily across the week and on weekdays, but not weekend days.

There were no significant interactions with gender in any model. Additional adjustments for BMI of participants and minutes of MVPA did not change the pattern of results (See Supplementary File A). Log-transformation of VPA did not change the pattern of results (See Supplementary File B).

For peer review only - http://bmjopen.bmj.com/site/about/guidelines.xhtml

(N = 5755)	Level	n (%)
Gender	Female	2888 (50.2)
	Male	2866 (49.8)
Country	England	4822 (83.7)
	Wales	269(4.7)
	Scotland	481 (8.4)
	Northern Ireland	182 (3.2)
Weight Status	Not overweight	4804 (83.8)
	Overweight	740 (12.9)
	Obese	190 (3.3)
Ethnic group	White	5289 (91.9)
	Mixed	142 (2.5)
	Indian	75 (1.3)
	Pakistani or Bangladeshi	105 (1.8)
	Black or Black British	92 (1.6)
	Other ethnic group	51 (0.9)
Maternal education	No qualifications	282 (4.9)
	Low	1416 (24.6)
	Medium	847 (14.7)
	High	2335 (40.6)
	Higher	509.4 (8.9)
Equivalised weekly household income (£), mean (SD)		459.4 (245.5)

Table 1. Socio-demographic characteristics of the weighted sample: UK Millennium Cohort Accelerometer Study, 4th follow-up (2008-09)

(N = 5755)	All days [1]	Weekday [1]	Weekend [1]
Mean time (mins/day) in sedentary behaviour	392.9 (64.1)	398.1 (66.4)	377.6 (88.3)
Mean time (mins/day) in light activity	281.8 (39.8)	281.5 (42.2)	282.7 (53.5)
Mean time (mins/day) in moderate activity	42.7 (12.9)	42.6 (13.4)	42.9 (18.8)
Mean time (mins/day) in vigorous activity	20.1 (10.7)	20.4 (11.3)	19.5 (14.1)
Mean time (mins/day) worn across all valid days	737.5 (59.0)	743.8 (61.8)	724.8 (85.2)

[1] All values are mean (SD)

Table 2. Physical activity characteristics of the weighted sample: UK Millennium Cohort Accelerometer Study, 4th follow-up (2008-09)

For peer review only

Exposure	Level	Mean mins vigorous physical activity/day[1], β-coefficient (lower, upper confidence intervals) Overall	Weekdays	Weekend days
Maternal education (ref: none)	Low	1.33 (0.32, 2.34)	1.37 (0.32, 2.41)	1.10 (-0.30, 2.51)
	Medium	1.57 (0.52, 2.62)	1.66 (0.56, 2.76)	1.14 (-0.30, 2.58)
	High	1.54 (0.58, 2.51)	1.61 (0.61, 2.60)	1.18 (-0.19, 2.54)
	Higher	2.84 (1.54, 4.13)	2.91 (1.56, 4.26)	2.47 (0.75, 4.20)
Equivalised Household Income	Per £10000/year	0.35 (0.12, 0.57)	0.33 (0.10, 0.56)	0.37 (0.10, 0.64)
Ethnic group (ref: white)	Mixed	1.87 (0.27, 3.46)	2.01 (0.23, 3.79)	0.98 (-0.53, 2.50)
	Indian	0.90 (-1.26, 3.07)	0.43 (-1.88, 2.73)	1.22 (-1.65, 4.09)
	Pakistani & Bangladeshi	-2.84 (-3.02, -1.77)	-2.95 (-4.14, -1.76)	-2.48 (-3.66, -1.30)
	Black or black British	-1.49 (-3.02, 0.03)	-1.77 (-3.45, -0.09)	-0.65 (-2.55, 1.25)
	Other ethnic group	-1.15 (-3.17, 0.87)	-1.66 (-3.91, 0.59)	-0.24 (-2.68, 2.19)

[1] All models are adjusted for moderate physical activity, season of measurement, wear time, age and sex

Table 3. Multivariable linear regression models for mean minutes of VPA overall, weekdays and weekend days, by socioeconomic and ethnic subgroups (n=5098) within the Millennium Cohort Accelerometer Study, 4th follow-up (2008-09)

For peer review only

DISCUSSION

This study reveals clear socioeconomic and ethnic differences in children's time spent in VPA. Children from less affluent backgrounds, alongside those from certain minority ethnic groups (Bangledeshi and Pakistani, Black or Black British), accumulated less of their daily activity from vigorous intensity activities. These differences were consistent in both boys and girls, and mirror existing inequalities in childhood adiposity.[40] Although the effect sizes are relatively small, we suggest that these differences are relevant at a population level and changes to reduce existing gaps in VPA could have population-level implications for inequalities in adiposity in UK children.

To our knowledge, this is the first study to investigate socio-economic and ethnicity-related differences in the patterning of children's VPA, accounting for time spent in MPA. While prior studies have investigated activity intensity,[40, 42] these did not account for MPA or have the capacity to investigate subgroups due to sample homogeneity. Accounting for MPA when analysing VPA is critical given differences in the accumulation and distribution of overall activity across individuals. MCS offers the largest available representative accelerometer dataset of UK children from all four countries. The objective measurement of free living activity using accelerometers is critical when comparing subgroups of children,[42] as self-reported data is likely to be differentially biased across these subgroups.[43] An additional strength of the cohort comes from the stratified sampling design which enabled adequate representation of socioeconomically disadvantaged and ethnic minority children. This is further enhanced by the comprehensive socio-demographic information linked through parent interviews. Much previous evidence relies on child-reported assessment of socio-economic position, resulting in substantial missing or invalid data, with higher rates of non-response from socioeconomically deprived children.[44]

Like any birth cohort, representativeness of the MCS sample is affected by participant attrition between waves of assessment. Prior analyses of MCS have demonstrated that boys, certain ethnic minorities (Indian, Pakistan/Bangladeshi and Black Caribbean/African), and children living with only one parent were less likely to provide valid accelerometer data.[45] Drop-out analyses showed that the participants included in this analysis were more likely to be male and come from a higher socioeconomic background. Although this may affect the representativeness of our findings, this is unlikely to have affected the associations observed. Lastly, accelerometers underestimate activity involving vertical movement (e.g. cycling) and those for which the accelerometer was not to be worn (e.g. aquatic activities or contact sports). If these behaviours are also socio-economically or ethnically patterned, this may have led to an under or overestimation of the true associations. More comprehensive measures of total physical activity, such as combining objective and self-report measures, may help overcome this bias.

There are multiple reasons why the differences in VPA observed may exist. Due to unequal access and costs, the organized contexts through which children accumulate VPA have been shown to

For peer review only - http://bmjopen.bmj.com/site/about/guidelines.xhtml

result in differences in participation between more and less advantaged subgroups of children.[23–25] These findings are however based on questionnaire-based assessments which capture sports and organized activity more accurately than other types of activity.[46] Additional factors, including parental perceptions of time commitments, and the limited variety of activities accessible, are significant factors linked to low levels of vigorous physical activity in low income families.[47] Furthermore, differences in home and family support for physical activity have been demonstrated between ethnic groups.[48] For instance, the presence of cultural and religious barriers have been found to impede participation in organized activity amongst Pakistani and Bangladeshi children, who in our analysis had the lowest levels of VPA. Our findings also demonstrate that differences in VPA between children from high and low maternal education families are more pronounced during the week. This may result from factors such as longer, inconsistent work hours within low-income jobs. These differences and differential barriers should be considered when developing interventions to increase VPA, and potentially impact obesity prevalence, in all groups. Furthermore, this finding highlights the potential of school-based and after-school sports clubs to contribute to a solution for reducing existing behavioural inequalities.

International physical activity guidelines for children focus on MVPA in part because increases in MVPA are hypothesised to be easier to achieve than VPA at a population level.[49] Our findings indicate that by utilizing the aggregate measure of MVPA we may be overlooking significant differences in the relative participation in MPA and VPA between population subgroups and tolerating substantial inequalities in the most important segment of physical activity for health outcomes. UK activity guidelines additionally recommend that children 'minimise the amount of time spent being sedentary for extended periods' and that 'vigorous intensity activities be incorporated at least three times a week'. Our results provide further empirical evidence to support the findings of Richards et al. (2015),[50] and their accompanying call to place more attention on the VPA component of guidelines to ensure health benefits. Further evidence suggests that childhood participation in sporting activities is vital to the development of fundamental motor skills, which strongly predict physical activity and weight status both in childhood and throughout adulthood.[51] To lay a foundation for lifelong activity participation it is critical that children, especially those from disadvantaged backgrounds, are provided with sufficient opportunities to develop fundamental motor skills. However, there is currently insufficient evidence concerning the most appropriate daily dose of VPA, and how to effectively promote VPA across population subgroups. Research efforts are needed to develop effective interventions for increasing VPA.

For peer review only - http://bmjopen.bmj.com/site/about/guidelines.xhtml

CONCLUSION

We found that the amount of vigorous intensity activity accumulated was socio-economically and ethnically patterned in 7-year-old UK children, mirroring known inequalities in adiposity.[40] These findings suggest that the current central focus of physical activity guidelines, and accordingly interventions, on the promotion of aggregate measures of MVPA, may be masking behavioural differences that may have an influential role in widening inequalities in obesity between more and less advantaged subgroups. In efforts to combat rising and widening childhood obesity rates, our results suggest a need for a greater focus on the promotion of VPA in health promotion efforts, particularly for those children from more disadvantaged backgrounds.

For peer review only

For peer review only - http://bmjopen.bmj.com/site/about/guidelines.xhtml

ACKNOWLEDGEMENTS

The co-operation of the participating families is gratefully acknowledged. The fourth sweep of the MCS was funded by grants to Professor Heather Joshi, former director of the study, from the Economic and Social Research Council and a consortium of government funders. The current director is Professor Lucinda Platt. The authors acknowledge the Centre for Longitudinal Studies, Institute of Education, for the use of these data; the UK Data Service for making them available; and the MRC Centre of Epidemiology for Child Health (grant reference G0400546), Institute of Child Health, University College London, for creating the accelerometer data resource, which was funded by the Wellcome Trust (grant reference 084686/ Z/08/A). The institutions and funders acknowledged bear no responsibility for the analysis or interpretation of these data. We also would like to thank Stephen Sharp at the MRC Epidemiology Unit for his statistical insights and input on the models.

AUTHOR'S CONTRIBUTIONS

RL and EvS designed the study. JA and AA provided inputs on preliminary results and contributed to redeveloping the statistical models. RL conducted all of the analyses and drafted the manuscript. All authors contributed to the interpretation of the results and critically reviewed the manuscript. All authors read and approved the final manuscript. In addition, Stephen Sharp, Senior Statistician at the MRC Epidemiology Unit provided advice on the statistical models. RL is the guarantor and responsible for the overall content.

For peer review only

References

1. Abarca-Gómez L, Abdeen ZA, Hamid ZA, et al. Worldwide trends in body-mass index, underweight, overweight, and obesity from 1975 to 2016: a pooled analysis of 2416 population-based measurement studies in 128·9 million children, adolescents, and adults. *Lancet*. 2017;0(0):1-16. doi:10.1016/S0140-6736(17)32129-3

2. Kelly T, Yang W, Chen C-S, Reynolds K, He J. Global burden of obesity in 2005 and projections to 2030. *Int J Obes (Lond)*. 2008;32(9):1431-1437. doi:10.1038/ijo.2008.102

3. Reilly J, Kelly J. Long-term impact of overweight and obesity in childhood and adolescence on morbidity and premature mortality in adulthood: systematic review. *Int J Obes*. 2010;35(10):891-898. doi:10.1038/ijo.2010.222

4. UK Government Department for International Development. Agenda 2030: Delivering the Global Goals. 2017;(March 2017):29 f. https://www.gov.uk/government/publications/agenda-2030-delivering-the-global-goals.

5. Goisis A, Sacker A, Kelly Y. Why are poorer children at higher risk of obesity and overweight ? A UK cohort study. 2015:1-7. doi:10.1093/eurpub/ckv219

6. Hargreaves DS, Marbini AD, Viner RM. Inequality trends in health and future health risk among English children and young people, 1999-2009. *Arch Dis Child*. 2013;98(11):850-855. doi:10.1136/archdischild-2012-303403

7. Shackleton N, Hale D, Viner RM. Trends and socioeconomic disparities in preadolescent's health in the UK: Evidence from two birth cohorts 32 years apart. *J Epidemiol Community Health*. 2015;70(2):140-146. doi:10.1136/jech-2015-205603

8. Martinson ML, Mclanahan S, Brooks-gunn J. Race/Ethnic and Nativity Disparities in Child Overweight in the United States and England. 2012;643(1):219-238. doi:10.1177/0002716212445750.Race/Ethnic

9. Zilanawala A, Davis-Kean P, Nazroo J, Sacker A, Simonton S, Kelly Y. Race/ethnic disparities in early childhood BMI, obesity and overweight in the United Kingdom and United States. *Int J Obes*. 2015;39(3):520-529. doi:10.1038/ijo.2014.171

10. Ekelund U. Moderate to Vigorous Physical Activity and Sedentary Time and Cardiometabolic Risk Factors in Children and Adolescents. *JAMA*. 2012;307(7):704. doi:10.1001/jama.2012.156

11. Janssen I, Katzmarzyk PT, Boyce WF, et al. Comparison of overweight and obesity prevalence in school-aged youth from 34 countries and their relationships with physical activity and dietary patterns. *Obes Rev*. 2005;6(2):123-132. doi:10.1111/j.1467-789X.2005.00176.x

12. Steinbeck KS. The importance of physical activity in the prevention of overweight and obesity in childhood: a review and an opinion. *Obes Rev*. 2001;2(2):117-130. doi:10.1046/j.1467-789x.2001.00033.x

13. Steele RM, van Sluijs EM, Sharp SJ, Landsbaugh JR, Ekelund U, Griffin SJ. An investigation of patterns of children's sedentary and vigorous physical activity throughout the week. *Int J Behav Nutr Phys Act*. 2010;7:88. http://ovidsp.ovid.com/ovidweb.cgi?T=JS&PAGE=reference&D=prem&NEWS=N&AN=21143901.

14. Pavey TG, Peeters G, Bauman AE, Brown WJ. Does Vigorous Physical Activity Provide Additional Benefits beyond Those of Moderate? *Med Sci Sport Exerc*. 2013;45(10):1948-

For peer review only - http://bmjopen.bmj.com/site/about/guidelines.xhtml

1955. doi:10.1249/MSS.0b013e3182940b91

15. Carson V, Rinaldi RL, Torrance B, et al. Vigorous physical activity and longitudinal associations with cardiometabolic risk factors in youth. 2013;(July):16-21. doi:10.1038/ijo.2013.135

16. Owens S, Galloway R, Gutin B. The case for vigorous physical activity in youth. *Am J Lifestyle Med*. 2017;11(2):96-115. doi:10.1177/1559827615594585

17. Gutin B, Zenong Y, Humphries M, Barbeau P. Relations of moderate and vigorous physical activity to fitness and fatness in adolescents. *Am J Clin Nutr*. 2005;(81):746-750.

18. Griffiths LJ, Sera F, Cortina-Borja M, Law C, Ness A, Dezateux C. Objectively measured physical activity and sedentary time: Cross-sectional and prospective associations with adiposity in the millennium cohort study. *BMJ Open*. 2016;6(4). doi:10.1136/bmjopen-2015-010366

19. Riddoch CJ, Mattocks C, Deere K, et al. Objective measurement of levels and patterns of physical activity. *Arch Dis Child*. 2007;92(11):963-969. doi:10.1136/adc.2006.112136

20. Ruiz JR, Ortega FB, Martínez-Gómez D, et al. Objectively measured physical activity and sedentary time in European adolescents: the HELENA study. *Am J Epidemiol*. 2011;174(2):173-184. doi:10.1093/aje/kwr068

21. Bammann K, Gwozdz W, Pischke C, et al. The impact of familial , behavioural and psychosocial factors on the SES gradient for childhood overweight in Europe . A longitudinal study. 2016;41(1):54-60. doi:10.1038/ijo.2016.137

22. Griffiths LJ, Cortina-Borja M, Sera F, et al. How active are our children? Findings from the Millennium Cohort Study. *BMJ Open*. 2013;3(8):e002893-e002893. doi:10.1136/bmjopen-2013-002893

23. Marques A, Ekelund U, Sardinha LB. Associations between organized sports participation and objectively measured physical activity, sedentary time and weight status in youth. *J Sci Med Sport*. 2015:2-5. doi:10.1016/j.jsams.2015.02.007

24. Fernandes R a, Reichert FF, Monteiro HL, et al. Characteristics of family nucleus as correlates of regular participation in sports among adolescents. *Int J Public Health*. 2012;57(2):431-435. doi:10.1007/s00038-010-0207-7

25. Hardy LL, Kelly B, Chapman K, King L, Farrell L. Parental perceptions of barriers to children's participation in organised sport in Australia. *J Paediatr Child Health*. 2010;46(4):197-203. doi:10.1111/j.1440-1754.2009.01661.x

26. Corder K, Sharp SJ, Atkin AJ, et al. Age-related patterns of vigorous-intensity physical activity in youth: The International Children's Accelerometry Database. *Prev Med Reports*. 2016;4:17-22. doi:10.1016/j.pmedr.2016.05.006

27. Hansen K, Jones E, Joshi H. *Millennium Cohort Study Fourth Survey : A User ' s Guide to Initial Findings*. London; 2010.

28. Studies C for L. *Millennium Cohort Study: A Guide to the Datasets (Eight Edition); First, Second, Third, Fourth and Fifth Surveys.* London; 2014.

29. Ott A, Pate R, Trost S, Ward D, Saunders R. The Use of Uniaxial and Triaxial Accelerometers to Measure Children's "Free-Play" Physical Activity. *Pediatr Exerc Sci*. 2000;12(4):360-370.

30. Trost S, Ward D, Moorehead S, Watson P, Riner W, Burke J. Validity of the computer science

For peer review only

and applications (CSA) activity monitor in children. *Med Sci Sport Exerc*. 1998;30(4):629-633.

31. Puyau MR, Adolph AL, Vohra FA, Butte NF. Validation and calibration of physical activity monitors in children. *Obes Res*. 2002;10(3):150-157. doi:10.1038/oby.2002.24

32. Marco Geraci A, Rich C, Sera F, Cortina-Borja M, Griffiths LJ. Accelerometry Data Processing Technical report on accelerometry data processing in the Millennium Cohort Study. 2012.

33. Mattocks C, Ness A, Leary S, et al. Use of accelerometers in a large field-based study of children: protocols, design issues, and effects on precision. *J Phys Act Health*. 2008;5 Suppl 1:S98-111. http://www.ncbi.nlm.nih.gov/pubmed/18364528. Accessed April 26, 2018.

34. Pillas D, Marmot M, Naicker K, Goldblatt P, Morrison J, Pikhart H. Social inequalities in early childhood health and development: A European-wide systematic review. *Pediatr Res*. 2014;76(5):418-424. doi:10.1038/pr.2014.122

35. Rosenberg R. *MCS4 : Guide to Derived Variables*.; 2012.

36. Statistics O for N. *Ethnic Group Statistics: A Guide for the Collection and Classification of Ethnicity Data*. London; 2003.

37. Fairclough SJ, Boddy LM, Mackintosh KA, Valencia-Peris A, Ramirez-Rico E. Weekday and weekend sedentary time and physical activity in differentially active children. *J Sci Med Sport*. 2015;18(4):444-449. doi:10.1016/j.jsams.2014.06.005

38. Griffiths L, Rich C, Geraci M, et al. *Technical Report on the Enhancement of Millennium Cohort Study Data with Accelerometer-Derived Measures of Physical Activity and Sedentary Behaviour in Seven Year Olds*.; 2013.

39. Cole T, Bellizzi M, Flegal K, Dietz W. Establishing a standard definition for child overweight and obesity worldwide: international survey. *Bmj*. 2000;320:1240-1243.

40. Brophy S, Cooksey R, Gravenor MB, et al. Risk factors for childhood obesity at age 5: analysis of the millennium cohort study. *BMC Public Health*. 2009;9:467. doi:10.1186/1471-2458-9-467

41. Wilkie HJ, Standage M, Gillison FB, Cumming SP, Katzmarzyk PT. Correlates of intensity-specific physical activity in children aged 9–11 years: a multilevel analysis of UK data from the International Study of Childhood Obesity, Lifestyle and the Environment. *BMJ Open*. 2018;8(2):e018373. doi:10.1136/bmjopen-2017-018373

42. Slootmaker SM, Schuit AJ, Chinapaw MJ, Seidell JC, van Mechelen W. Disagreement in physical activity assessed by accelerometer and self-report in subgroups of age, gender, education and weight status. *Int J Behav Nutr Phys Act*. 2009;6:17. http://ovidsp.ovid.com/ovidweb.cgi?T=JS&PAGE=reference&D=prem&NEWS=N&AN=19320985.

43. Prince SA, Adamo KB, Hamel M, Hardt J, Connor Gorber S, Tremblay M. A comparison of direct versus self-report measures for assessing physical activity in adults: a systematic review. *Int J Behav Nutr Phys Act*. 2008;5(1):56. doi:10.1186/1479-5868-5-56

44. Wardle J, Robb K, Johnson F. Assessing socioeconomic status in adolescents: the validity of a home affluence scale. 2002:595-599.

45. Rich C, Cortina-Borja M, Dezateux C, et al. Predictors of non-response in a UK-wide cohort study of children's accelerometer-determined physical activity using postal methods. *BMJ*

For peer review only - http://bmjopen.bmj.com/site/about/guidelines.xhtml

Open. 2013;3(3). doi:10.1136/bmjopen-2012-002290

46. Corder K, Sluijs EMF Van, Wright A, Whincup P, Wareham NJ, Ekelund U. Is it possible to assess free-living physical activity and energy expenditure in young people by self-report ? 1 – 3. 2009:862-870. doi:10.3945/ajcn.2008.26739.862

47. Brophy S, Cooksey R, Lyons RA, Thomas NE, Rodgers SE, Gravenor MB. Parental factors associated with walking to school and participation in organised activities at age 5: analysis of the Millennium Cohort Study. *BMC Public Health*. 2011;11(1):14. doi:10.1186/1471-2458-11-14

48. Trigwell J, Murphy RC, Cable NT, Stratton G, Watson PM. Parental views of children ' s physical activity : a qualitative study with parents from multi-ethnic backgrounds living in England. 2015:1-11. doi:10.1186/s12889-015-2351-8

49. Tarp J, Child A, White T, et al. Physical activity intensity, bout-duration, and cardiometabolic risk markers in children and adolescents. *Int J Obes*. 2018:1-12. doi:10.1038/s41366-018-0152-8

50. Richards J, Doherty A, Foster C. Is the Current Focus of the Global Physical Activity Recommendations for Youth Appropriate in All Settings ? and Cardiorespiratory Fitness Patterns for Current Global Physical Activity. :901-903.

51. Lubans DR, Morgan PJ, Cliff DP, Barnett LM, Okely AD. Fundamental Movement Skills in Children and Adolescents Review of Associated Health Benefits. *Sport Med*. 2010;40(12):1019-1035.

For peer review only

For peer review only

Supplementary Files

Supplementary File A: Additional model adjustments

Multivariable linear regression models for mean mins of vigorous physical activity (VPA) for all valid days adjusted for (1) MPA & wear time; (2) MPA, wear time & BMI; (3) MVPA & wear time, by socioeconomic and ethnic subgroups

Equity subgroups	Mean mins of vigorous physical activity (VPA) (all valid days) β-coef (LCI, UCI)			
Socioeconomic Position	**Maternal education**			
	[Reference cat: No qualifications]	Adjusted for MPA, wear time, age and sex [1]	Adjusted for MPA, wear time, age, sex & BMI [2]	Adjusted for MVPA, wear time, age and sex [3]
	Low	1.35 (0.34, 2.36)	1.37 (-0.01, 0.00)	0.75 (0.12, 1.38)
	Medium	1.62 (0.57, 2.67)	0.67 (0.14, 1.20)	0.94 (0.29, 1.60)
	High	1.58 (0.61, 2.54)	1.48 (0.52, 2.44)	0.90 (0.29, 1.51)
	Highest	2.88 (1.58, 4.17)	2.79 (1.49, 4.08)	1.67 (0.93, 2.42)
	**OECD Equivalised Household Income**			
	[Reference cat: No qualifications]	Adjusted for MPA & Wear time	Adjusted for MPA, wear time & BMI	Adjusted for MVPA & wear time
	[per £10 000 annual increase]	0.34 (0.12, 0.57)	0.30 (0.08, 0.53)	0.26 (0.14, 0.38)
Ethnicity	**Ethnic Group**			
	[Reference cat: White]	Adjusted for MPA & Wear time	Adjusted for MPA, wear time & BMI	Adjusted for MVPA & wear time
	Mixed	1.80 (0.21, 3.40)	1.73 (0.18, 3.29)	1.11 (0.18, 2.04)
	Indian	0.84 (-1.33, 3.01)	0.70 (-1.47, 2.88)	1.05(-0.18, 2.29)
	Pakistani & Bangladeshi	-2.89 (-3.96, -1.82)	-3.03 (-4.07, -2.00)	-1.59 (-2.28, -0.91)
	Black or black british	-1.52 (-3.05, 0.00)	-0.75 (-2.24, 0.73)	-0.84 (-1.74, 0.05)
	Other ethnic group	-1.14 (-3.16, 0.90)	-1.50 (-3.58, 0.57)	-0.42 (-1.76, 0.92)

For peer review only - http://bmjopen.bmj.com/site/about/guidelines.xhtml

Supplementary File B: Main models with log transformed mean daily minutes of vigorous intensity physical activity (VPA)

Multivariable linear regression models for mean mins of vigorous physical activity (VPA) for all valid days by socioeconomic and ethnic subgroups

Equity subgroups		Mean mins of log transformed vigorous physical activity (VPA) β-coef (LCI, UCI)
Socioeconomic Position	**Maternal education**	
	[Reference cat: No qualifications]	
	Low	0.06 (0.00, 0.11)
	Medium	0.08 (0.02, 0.14)
	High	0.09 (0.03, 0.14)
	Highest	0.13 (0.07, 0.19)
	**OECD Equivalised Household Income**	
	[per £10000 annual increase]	0.02 (0.01, 0.03)
Ethnicity	**Ethnic Group**	
	[Reference cat: White]	
	Mixed	0.10 (0.03, 0.17)
	Indian	0.02 (-0.10, 0.14)
	Pakistani & Bangladeshi	-0.16 (-0.22, -0.09)
	Black or black british	-0.06 (-0.13 – 0.02)
	Other ethnic group	-0.05 (-0.16, 0.06)

All models are adjusted for MPA and wear time

P-value: (* $p \leq 0.05$; ** $p \leq 0.01$; *** $p \leq 0.001$)

Acronyms: VPA (vigorous intensity physical activity); MPA (moderate intensity physical activity); LCI (lower confidence interval); UCI (upper confidence interval); NVQ (National Vocational Qualification); OECD (Organisation for Economic Cooperation and Development)

For peer review only

STROBE Statement—Checklist of items that should be included in reports of ***cross-sectional studies***

	Item No	Recommendation	Page No
**Title and abstract**	1	(*a*) Indicate the study's design with a commonly used term in the title or the abstract	1
		(*b*) Provide in the abstract an informative and balanced summary of what was done and what was found	3
**Introduction**			
Background/rationale	2	Explain the scientific background and rationale for the investigation being reported	5
Objectives	3	State specific objectives, including any prespecified hypotheses	5-6
**Methods**			
Study design	4	Present key elements of study design early in the paper	7-8
Setting	5	Describe the setting, locations, and relevant dates, including periods of recruitment, exposure, follow-up, and data collection	7
Participants	6	(*a*) Give the eligibility criteria, and the sources and methods of selection of participants	7
Variables	7	Clearly define all outcomes, exposures, predictors, potential confounders, and effect modifiers. Give diagnostic criteria, if applicable	7-8
Data sources/ measurement	8*	For each variable of interest, give sources of data and details of methods of assessment (measurement). Describe comparability of assessment methods if there is more than one group	7-8
Bias	9	Describe any efforts to address potential sources of bias	9
Study size	10	Explain how the study size was arrived at	7
Quantitative variables	11	Explain how quantitative variables were handled in the analyses. If applicable, describe which groupings were chosen and why	8
Statistical methods	12	(*a*) Describe all statistical methods, including those used to control for confounding	8
		(*b*) Describe any methods used to examine subgroups and interactions	8
		(*c*) Explain how missing data were addressed	8
		(*d*) If applicable, describe analytical methods taking account of sampling strategy	8
		(*e*) Describe any sensitivity analyses	8
**Results**			
Participants	13*	(a) Report numbers of individuals at each stage of study—eg numbers potentially eligible, examined for eligibility, confirmed eligible, included in the study, completing follow-up, and analysed	9
		(b) Give reasons for non-participation at each stage	9
		(c) Consider use of a flow diagram	-
Descriptive data	14*	(a) Give characteristics of study participants (eg demographic, clinical, social) and information on exposures and potential confounders	10
		(b) Indicate number of participants with missing data for each variable of interest	9
Outcome data	15*	Report numbers of outcome events or summary measures	11
Main results	16	(*a*) Give unadjusted estimates and, if applicable, confounder-adjusted estimates and their precision (eg, 95% confidence interval). Make clear which confounders were adjusted for and why they were included	11

For peer review only

For peer review only - http://bmjopen.bmj.com/site/about/guidelines.xhtml

		(*b*) Report category boundaries when continuous variables were categorized	11
		(*c*) If relevant, consider translating estimates of relative risk into absolute risk for a meaningful time period	-
Other analyses	17	Report other analyses done—eg analyses of subgroups and interactions, and sensitivity analyses	9
**Discussion**			
Key results	18	Summarise key results with reference to study objectives	12
Limitations	19	Discuss limitations of the study, taking into account sources of potential bias or imprecision. Discuss both direction and magnitude of any potential bias	12
Interpretation	20	Give a cautious overall interpretation of results considering objectives, limitations, multiplicity of analyses, results from similar studies, and other relevant evidence	13
Generalisability	21	Discuss the generalisability (external validity) of the study results	13
**Other information**			
Funding	22	Give the source of funding and the role of the funders for the present study and, if applicable, for the original study on which the present article is based	2

*Give information separately for exposed and unexposed groups.

Note: An Explanation and Elaboration article discusses each checklist item and gives methodological background and published examples of transparent reporting. The STROBE checklist is best used in conjunction with this article (freely available on the Web sites of PLoS Medicine at http://www.plosmedicine.org/, Annals of Internal Medicine at http://www.annals.org/, and Epidemiology at http://www.epidem.com/). Information on the STROBE Initiative is available at www.strobe-statement.org.

For peer review only - http://bmjopen.bmj.com/site/about/guidelines.xhtml

BMJ Open

Socio-economic and ethnic differences in children's vigorous intensity physical activity: a cross-sectional analysis of the UK Millennium Cohort Study

Journal:	*BMJ Open*
Manuscript ID	bmjopen-2018-027627.R1
Article Type:	Research
Date Submitted by the Author:	28-Feb-2019
Complete List of Authors:	Love, Rebecca; University of Cambridge Department of Public Health and Primary Care, UKCRC Centre for Diet and Activity Research (CEDAR), MRC Epidemiology Unit<br>Adams, J; University of Cambridge, Centre for Diet & Activity Research<br>Atkin, Andrew; University of East Anglia Faculty of Medicine and Health Sciences,<br>van Sluijs, Esther; University of Cambridge, MRC Epidemiology Unit
**Primary Subject Heading**:	Epidemiology
Secondary Subject Heading:	Public health
Keywords:	PUBLIC HEALTH, PREVENTIVE MEDICINE, Health policy < HEALTH SERVICES ADMINISTRATION & MANAGEMENT

SCHOLARONE™
Manuscripts

Title:

Socio-economic and ethnic differences in children's vigorous intensity physical activity: a cross-sectional analysis of the UK Millennium Cohort Study

Author names:

Rebecca Love (PhD Candidate), Jean Adams (Senior University Lecturer), Andrew J. Atkin (Lecturer in Behavioural Epidemiology), Esther MF van Sluijs (MRC Programme Leader)

Author affiliations:

MRC Epidemiology Unit and UKCRC Centre for Diet and Activity Research (CEDAR), University of Cambridge School of Clinical Medicine, Institute of Metabolic Science, Cambridge Biomedical Campus, Cambridge, UK

- (Rebecca Love, MSc; Jean Adams, PhD; Esther MF van Sluijs, PhD)

School of Health Sciences, Faculty of Medicine and Health Sciences, University of East Anglia, Norwich Research Park, Norwich NR4 7TJ, UK

- (Andrew J. Atkin, PhD)

Corresponding author:

Rebecca Love

MRC Epidemiology Unit & Centre for Diet and Activity Research (CEDAR)

University of Cambridge School of Clinical Medicine,

Box 285, Institute of Metabolic Science,

Cambridge Biomedical Campus,

Cambridge

CB2 0QQ

E-mail: rel54@medschl.cam.ac.uk

Telephone: 0122 3746877

Author email addresses:

Jean Adams: jma79@medschl.cam.ac.uk

Esther van Sluijs: esther.vansluijs@mrc-epid.cam.ac.uk

Andrew J Atkin: A.Atkin@uea.ac.uk

Word count:

For peer review only

For peer review only - http://bmjopen.bmj.com/site/about/guidelines.xhtml

3151

Competing interests:

All authors have no competing interests to declare.

Funding:

Funding for this study and the work of all authors was supported, wholly or in part, by the Centre for Diet and Activity Research (CEDAR), a UKCRC Public Health Research Centre of Excellence (087636/Z/08/Z; ES/G007462/1; MR/K023187/1). Funding from the British Heart Foundation, Department of Health, Economic and Social Research Council, Medical Research Council, and the Wellcome Trust, under the auspices of the UK Clinical Research Collaboration, is gratefully acknowledged. Rebecca Love is funded by a Gates Cambridge Scholarship. The work of Esther M F van Sluijs was supported by the Medical Research Council (MC_UU_12015/7). No funders were involved in the production of this manuscript.

Data sharing statement:

This is a secondary data analysis based on the Millennium Cohort Study data. The data and access policy is available online here:
https://beta.ukdataservice.ac.uk/datacatalogue/series/series?id=2000031#!/access

For peer review only

ABSTRACT

Objective: To investigate if daily vigorous physical activity, adjusted for minutes of moderate physical activity performed, differs by socio-economic position or ethnicity in a large sample of UK children with objectively measured physical activity.

Design: Nationally representative prospective cohort study.

Setting: UK children born between 2000 and 2002.

Participants: 5172 7-8-year-old children with valid accelerometer data for ≥10 h on ≥3 days, including one weekend day.

Main outcome measures: Time spent in vigorous physical activity (VPA) (>3841 counts per minute).

Explanatory measures: Maternal education, annual household OECD equivalised income, ethnicity.

Results: Multivariable linear regression models fitted to explore differences in average daily minutes of VPA (adjusted for moderate physical activity (MPA), mean accelerometer wear time, season of measurement, age and sex), revealed significantly higher amounts of VPA accumulated as a child's socioeconomic position increased (highest versus lowest level of maternal education: β: 2.96, p: 0.00; annual household equivalised income: β: 0.58, p: 0.00, per £10,000 annual increase). Additionally, children from certain minority ethnicities (Bangladeshi and Pakistani: β: -3.34, p: 0.00; Other ethnic groups: β:-2.27, p: 0.02) accrued less daily VPA compared with their white British counterparts.

Conclusions: The socioeconomic and ethnic patterning of vigorous activity observed in this study mirrors parallel inequalities in rates of childhood obesity. Given the stronger association of vigorous activity (VPA) with adiposity than of moderate activity (MPA), intensity specific differences may be contributing to widening inequalities in obesity. Accordingly, these findings suggest that the current global focus on overall MVPA may mask important behavioural inequalities.

For peer review only

For peer review only - http://bmjopen.bmj.com/site/about/guidelines.xhtml

STRENGTHS AND WEAKNESSES

- This is the first study to investigate socio-economic and ethnicity-related differences in children's vigorous intensity physical activity behaviour, accounting for moderate physical activity.

- We used data from the Millennium Cohort Study which used a stratified sampling design enabling adequate representation of socioeconomically disadvantaged and ethnic minority children.

- This is the largest available accelerometer dataset of objectively measured physical activity in UK children.

- Boys, certain ethnic minorities (Indian, Pakistan/Bangladeshi and Black Caribbean/African), and children living with only one parent were less likely to provide valid accelerometer data which may have affected the representativeness of our findings.

- The accelerometer measurements utilized are limited in their ability to classify behaviour, detect certain activities (e.g. cycling and swimming), upper body movements or changes in terrain.

For peer review only

BACKGROUND

Over the past four decades, global prevalence of childhood obesity has increased tenfold.[1] Predictions forecast that if current trends continue, by 2030, 60% of the global adult population will be overweight or obese.[2] Given that obesity in childhood is associated with premature mortality and physical morbidity in adulthood,[3] tackling childhood obesity is increasingly a public health priority for governments.[4]

Of increasing concern are widening inequalities in obesity prevalence. By age 11, UK children from lower income families are three times as likely to be obese than their more advantaged counterparts.[5] These differences progressively worsen with age, fuelling inequality in health across the life-course.[6,7] There are also stark ethnic and racial differences in childhood adiposity, with higher rates of obesity within certain ethnic minorities including Black British and Bangladeshi.[8,9] It is suggested that these differences are partially linked to existing disparities in socioeconomic resources between ethnic groups.[9] At present, there is limited understanding of the modifiable factors driving socio-economic and ethnicity-related disparities in childhood obesity.

Physical activity is a key behavioural driver of cardiovascular disease risk in children.[10] Evidence of an association with obesity is less clear.[11,12] However, consistent evidence suggests that vigorous intensity activity (VPA) is more strongly associated with reduced waist circumference and adiposity relative to lower intensity activity, including moderate intensity activity (MPA).[13–17]

Previous research using objectively measured physical activity has demonstrated no socioeconomic patterning in children's adherence to the international guidelines to engage in 'moderate-to-vigorous intensity activity (MVPA) for at least 60 minutes per day'.[18–20] While there is some evidence of ethnic differences in activity levels,[21] these analyses are confined to the use of aggregated MVPA to quantify activity levels. This focus on MVPA may be why physical activity only explains a small portion of the socioeconomic gradient in overweight risk present within UK children.[22]

The importance of intensity specific differences of MVPA in explaining socioeconomic and ethnic inequalities in health remains underexplored. Considering that moderate physical activity (MPA) and vigorous physical activity (VPA) are accumulated through different types of activities (e.g. walking to school vs. sport participation),[23] they may be differently distributed in population subgroups. In high-income countries including the UK, children's participation in the organized sports activities that drive VPA have been shown to be socially patterned due to unequal access, support and costs.[24–26] Children from certain ethnic minorities have been shown to face additional barriers to sport participation due to cultural and religious factors, lack of access and parental safety concerns.[27] The need for understanding of these intensity differences is strengthened by evidence suggesting that the age-related decreases in VPA observed annually into adulthood are more pronounced in non-white and socio-economically deprived children, than their counterparts.[28] We propose that socio-economic

For peer review only - http://bmjopen.bmj.com/site/about/guidelines.xhtml

84 and ethnic differences in children's higher activity intensity could help to explain well established
85 socio-economic and ethnic gradients in obesity. Thus, the objective of this study was to investigate if
86 daily VPA, adjusted for minutes of MPA performed, differs by socioeconomic position or ethnicity in
87 a large sample of UK children aged 7 years.

For peer review only

METHODS

Sample

This analysis used data from the Millennium Cohort Study (MCS), a nationally representative, longitudinal study of children born in the United Kingdom between September 2000 and January 2002.[29] MCS was developed to track the social, economic and health experiences facing children born at the start of the 21st century. The original cohort included 18,818 children. Data was first collected when cohort participants were 9 months old through a home-based interview (72% of those initially contacted provided information). Subsequent follow-up interviews at ages 3, 5, 7 and 11 years were conducted in the home environment with the main respondent (primarily the natural mother, and where applicable the partner).[30] At age 7 an accelerometer study was conducted with participants. Parental consent and child assent was obtained for participation in the accelerometer study

To ensure adequate representation of all four UK countries, including disadvantaged and minority populations, a stratified clustered sampling design was used to oversample children living in Wales, Scotland and Northern Ireland, disadvantaged areas, and communities with high proportions of minority ethnic groups. The MCS received ethics approval from the South West and London Multi-centre Research Ethnics Committees, UK and the Yorkshire Research Ethics Committee. The analyses presented here used data from follow-up 4 at age 7 years (MCS4).

Patient and Public Involvement

This analysis is of secondary data collected a decade ago and accordingly the participants included were not involved in the conceptualization or design of our specific study.

Physical Activity

At MCS4 participating children (n=13 681) were invited to participate in an accelerometer study. Consenting children were sent a pre-programmed Actigraph GT1M accelerometer (Actigraph, Pensacola, Florida), set to record data at 15-second epochs. All data collection and processing was performed in-house, according to predetermined criteria.[31]

Actigraph accelerometers have been shown to be reliable and valid in comparison to measures of physical activity derived from heart rate monitoring, indirect and room calorimetry, and doubly labelled water.[32–34] Children were instructed to wear the accelerometer on an elastic belt around their waist for 7 days throughout all waking hours (except during aquatic activities or contact sports), and to return it by post. Children were additionally asked to complete a monitor wear log to help identify non-wear time. Data was collected over a 15-month period between May 2008 – August 2009.

Data was downloaded using Actigraph software V.3.8.3 (Actigraph, Pensacola, Florida, USA) and processed according to predetermined criteria, using the R statistical package pawacc.[31] Non-wear

For peer review only

time, defined as 20 minutes or more of consecutive zero activity counts, were excluded from the analysis. Counts were separated into sedentary (<100 counts per min (cpm)), moderate (2240-3840 cpm) and vigorous (>3841 cpm) categories.[31] To ensure reliable estimates of activity, the sample was restricted to participants with 3 valid days of data (≥ 10 h/day, including at least 1 weekend day).[35] The sample was additionally restricted to singleton children. Extreme values above a threshold of ≥11715 cpm were excluded, as this may indicate a faulty monitor.

Sociodemographic variables

Considering strong evidence that health and obesity is patterned strongly and independently along both socioeconomic [36–38] and ethnic [39,40] lines, our analysis examines the association with both indicators. Information pertaining to socioeconomic position and ethnicity were collected at MCS4. Socio-economic position was measured using maternal education and equivalised household income. Maternal education captures the socioeconomic circumstances that accrue to a child and is advantageous as it can be applied to mothers irrespective of whether or not they are in paid employment at the time of interview.[41] Maternal education was categorized into five groups: none (qualifications less than those currently expected when leaving school at 16 years); low (qualifications comparable to those currently expected when leaving school at 16 years); medium (qualifications comparable to those currently expected when leaving school at 18 years); high (qualifications greater than medium, but not higher); and higher (any higher educational qualifications).[42] Annual household income was equivalized for household composition based on guidance from the Organisation for Economic Co-Operation and Development (OECD).[29] Ethnicity was parent-reported and categorised in our analyses as: white, any mixed, Indian, Pakistani or Bangladeshi, black or black British, or other. Parents were asked to select from a longer list which ethnic group they identify most with (E.g. Black includes those who identified as Black Caribbean, Black African and Black British). The ethnic classifications utilized were based on census categories in accordance with guidelines from the Office for National Statistics.[43]

Statistical analysis

Multivariable linear regression models were fitted to analyse differences in absolute mean daily minutes of VPA achieved across socio-economic and ethnic groups, adjusting for mean daily minutes of MPA, mean accelerometer wear time, season of measurement, age and sex. Separate models were run for each exposure variable (maternal education, equivalised household income, ethnicity) to assess the effects of each seperately.[44] Models were also run separately for week and weekend days as there is evidence that children accumulate physical activity differently on weekdays and weekend days.[45] Further, it is possible that different socio-economic and ethnic subgroups of children engage in different types of activities with distinct weekly patterning (e.g. weekend sports). All model residuals were assessed for normality. To investigate effect modification by gender,

For peer review only - http://bmjopen.bmj.com/site/about/guidelines.xhtml

interactions were run across all models. In sensitivity analyses, additional adjustments for BMI were explored. All analyses were conducted using STATA 15.1 software, with survey commands used to account for the stratified clustered design of MCS and to obtain robust standard errors.[46,47]. Sampling weights adjusted for unit nonresponse between waves were utilized.

To support the premise of the current analyses that VPA is most strongly associated with adiposity, additional linear regression models were fitted to study differences in BMI-zscore by mean daily minutes of VPA and MPA separately, adjusting for accelerometer wear time, age and sex.

For peer review only

RESULTS

Of the 12,872 children that consented to the accelerometer study, 9,772 returned the accelerometers, with a final sample of 6,497 children following in-house processing by MCS.[48] Application of our study inclusion criteria resulted in an analytic sample of 5,172 children. This drop was predominately driven by our requirement participant's '3 or more valid days', include one weekend day to enable comparisons across weekend and weekdays. On average, children in the weighted sample were 6.8 years of age (SD: 0.4) and 50% female (See Table 1). Overall, 14.4% of girls and 11.7% of boys were overweight, whilst 4.1% and 3.5% were obese. These classifications were made through application of the World Health Organization Growth Standards to produce age and gender specific zscores utilizing Stata functions zanthro and zbmicat.[49] The sample included children from each country across the United Kingdom (England, Wales, Scotland and Northern Ireland). Socio-demographic and physical activity summary characteristics of the analytic sample are outlined in Table 1 and 2, respectively. These two tables of sample characteristics are based on the weighted sample given that all our analyses are weighted. Drop out analyses showed that those participants included in the analytic sample (n=5172) were more likely to come from a higher income household, have mothers with higher levels of education, and be male when compared to participants who provided accelerometer data but did not meet the criteria for the analytic sample (n=1325).

Multivariable linear regression models revealed significant differences in the minutes of daily VPA accumulated across socio-economic subgroups (Table 3, see supplementary file 1 for full model details). Significantly more minutes of daily VPA was accumulated in each level of maternal education compared to those whose mother indicated 'no qualifications'. This relationship was more pronounced on weekdays than on weekend days. Analyses by equivalized household income supported this, indicating significantly more time spent in VPA with increasing household income. Figure 1 illustrates this effect and shows the proportion of VPA within daily MVPA by categories of activity, stratified into tertiles of equivalised household income. This demonstrates that irrespective of activity level, children from higher affluence families generally accumulate a greater proportion of their daily MVPA from VPA.

Pakistani & Bangladeshi children performed on average over 3 minutes less daily VPA in comparison to white British children overall, on weekdays and weekend days. This difference was slightly more pronounced on weekdays, versus weekend days. Children from 'other ethnic groups' also accumulated less daily VPA overall and on weekdays (2.2 and 3 minutes less, respectively). In contrast, children from a mixed ethnic descent accumulated comparatively more minutes of VPA daily across the week and on weekdays, but not weekend days.

There were no significant interactions with gender in any model. Additional adjustments for BMI z-score did not change the pattern of results (See supplementary file 2). Supporting multivariable

For peer review only

For peer review only - http://bmjopen.bmj.com/site/about/guidelines.xhtml

linear regression models for BMI z-score revealed a significant association between daily minutes of VPA and BMI z-score, with a 1-minute difference in VPA associated with 0.012 lower BMI z-score (See Supplementary File 3). The association of daily MPA with BMI z-score was statistically significant, but substantially smaller (-0.002).

For peer review only

For peer review only - http://bmjopen.bmj.com/site/about/guidelines.xhtml

Table 1. Socio-demographic characteristics of the weighted sample: UK Millennium Cohort Accelerometer Study, 4th follow-up (2008-09)

(N = 5172)	Level	n (%)
Gender	Female	2592 (50.1)
	Male	2580 (49.8)
Country	England	3390 (65.5)
	Wales	676 (13.1)
	Scotland	612 (11.8)
	Northern Ireland	494 (9.6)
Weight Status	Not overweight	4296 (83.3)
	Overweight	663 (12.9)
	Obese	196 (3.8)
Ethnic group	White	4543 (87.8)
	Mixed	130 (2.5)
	Indian	115 (2.2)
	Pakistani or Bangladeshi	194 (3.8)
	Black or Black British	127 (2.5)
	Other ethnic group	63 (1.2)
Maternal education	No qualifications	263 (5.1)
	Low	1241 (24.0)
	Medium	787 (15.2)
	High	1965 (38.0)
	Higher	447 (8.6)
	Overseas qual.	108 (2.1)
	None of these	361 (7.0)
Equivalised monthly household income (£), mean (SD)		1700.9 (943.1)
Distribution of valid days		6.1 (1.2)
Age (years)		6.8 (0.39)

For peer review only

Table 2. Physical activity characteristics of the weighted sample: UK Millennium Cohort Accelerometer Study, 4th follow-up (2008-09

(N = 5172)	All days [1]	Weekday [1]	Weekend [1]
Mean time (mins/day) in sedentary behaviour	397.2 (68.3)	402.8 (70.8)	380.5 (91.9)
Mean time (mins/day) in light activity	282.7 (40.5)	282.9 (42.9)	282.6 (54.5)
Mean time (mins/day) in moderate activity	42.5 (13.2)	42.4 (13.6)	42.9 (19.1)
Mean time (mins/day) in vigorous activity	19.9 (10.6)	20.1 (11.1)	19.4 (14.2)
Mean time (mins/day) worn across all valid days	742.4 (63.1)	748.2 (66.1)	725.4 (88.3)

[1] All values are mean (SD)

For peer review only

Table 3. Multivariable linear regression models for mean minutes of VPA and MPA respectively, overall for all valid days, on weekdays and on weekend days, by socioeconomic and ethnic subgroups (n=5172) within the Millennium Cohort Accelerometer Study, 4th follow-up (2008-09)

		Overall			Weekdays			Weekends		
Exposure	Level	β-coeff	[95% Conf	Interval]	β-coef	[95% Conf	Interval]	β-coef	[95% Conf	Interval]
VPA minutes/week [1]										
Ethnic group (ref: white)	Mixed	1.47*	-0.06	3.00	1.57*	-0.14	3.28	0.43	-1.42	2.28
	Indian	0.74	-1.19	2.67	0.58	-1.23	2.39	0.76	-2.18	3.70
	Pakistani & Bangladeshi	-3.34***	-4.66	-2.03	-3.45***	-4.91	-1.99	-3.00***	-4.36	-1.64
	Black or Black British	-1.67	-3.96	0.61	-2.08	-4.47	0.32	-0.03	-3.44	3.37
	Other ethnic group	-2.27**	-4.16	-0.37	-3.07***	-5.31	-0.83	-0.14	-2.28	2.00
Maternal education (ref:none)	Low	1.31**	0.22	2.41	1.31**	0.11	2.52	1.34*	-0.06	2.73
	Medium	1.65***	0.53	2.77	1.72***	0.49	2.95	1.37*	-0.08	2.81
	High	1.81***	0.77	2.86	1.88***	0.73	3.03	1.63**	0.32	2.94
	Higher	2.96***	1.45	4.46	3.04***	1.39	4.70	2.80***	1.06	4.53
	Overseas qual.	2.28*	-0.30	4.85	2.12	-0.45	4.70	2.69	-0.67	6.05
	None of these	-0.45	-1.77	0.87	-0.60	-2.02	0.83	-0.07	-1.77	1.64
Equivalised income	Per £ 10,000/year	0.58***	0.32	0.84	0.61***	0.35	0.88	0.50***	0.18	0.82
MPA minutes/week [2]										
Ethnic group (ref: white)	Mixed	-2.22**	-4.25	-0.19	-2.00*	-4.23	0.23	-2.13	-5.04	0.78
	Indian	-3.94***	-6.23	-1.65	-3.46***	-5.63	-1.30	-4.46**	-7.96	-0.96
	Pakistani & Bangladeshi	2.09**	0.33	3.85	2.00**	0.11	3.89	2.02*	-0.33	4.37
	Black or Black British	3.01***	1.07	4.95	3.10***	0.81	5.39	1.77	-0.96	4.49
	Other ethnic group	1.09	-2.30	4.47	1.89	-1.82	5.59	-1.34	-5.37	2.70
Maternal education (ref:none)	Low	-0.95	-2.59	0.69	-0.94	-2.69	0.81	-1.32	-3.38	0.75
	Medium	-1.48*	-3.15	0.19	-1.38	-3.16	0.39	-1.89*	-4.07	0.29
	High	-1.69**	-3.29	-0.09	-1.73**	-3.44	-0.03	-1.71*	-3.70	0.28
	Higher	-2.74***	-4.55	-0.92	-2.71***	-4.59	-0.82	-3.56***	-5.90	-1.22
	Overseas qual.	-1.96	-4.57	0.66	-2.29	-5.05	0.46	-1.74	-5.56	2.09
	None of these	1.98*	-0.05	4.01	1.88*	-0.31	4.06	2.35*	-0.31	5.00
Equivalised hincome	Per £ 10,000/year	-0.98***	-1.25	-0.70	-0.99***	-1.27	-0.71	-0.92***	-1.33	-0.50

[1] All models are adjusted for moderate physical activity, season of measurement, wear time, age and sex

[2] All models are adjusted for vigorous physical activity, season of measurement, wear time, age and sex

For peer review only - http://bmjopen.bmj.com/site/about/guidelines.xhtml

For peer review only

DISCUSSION

This study reveals clear socioeconomic and ethnic differences in children's time spent in VPA. Children from less affluent backgrounds, alongside those from certain minority ethnic groups (Bangledeshi and Pakistani, Other ethnic groups), accumulated less of their daily activity from vigorous intensity activities. These differences were consistent in both boys and girls, and mirror existing inequalities in childhood adiposity.[50] Although the effect sizes are relatively small, and possibly not clinically relevant at an individual level, we suggest that these differences are relevant at a population level and changes to reduce existing gaps in VPA could have population-level implications for inequalities in adiposity in UK children.[10]

To our knowledge, this is the first study to investigate socio-economic and ethnicity-related differences in the patterning of children's VPA, accounting for time spent in MPA. While prior studies have investigated activity intensity,[51] these did not account for MPA or have the capacity to investigate subgroups due to sample homogeneity. Accounting for MPA when analysing VPA is critical given differences in the accumulation and distribution of overall activity across individuals. MCS offers the largest available representative accelerometer dataset of UK children from all four countries. The objective measurement of free living activity using accelerometers is critical when comparing subgroups of children,[52] as self-reported data is likely to be differentially biased across these subgroups.[53] An additional strength of the cohort comes from the stratified sampling design which enabled adequate representation of socioeconomically disadvantaged and ethnic minority children. This is further enhanced by the comprehensive socio-demographic information linked through parent interviews. Much previous evidence relies on child-reported assessment of socio-economic position, resulting in substantial missing or invalid data, with higher rates of non-response from socioeconomically deprived children.[54]

Like any birth cohort, representativeness of the MCS sample is affected by participant attrition between waves of assessment. Prior analyses of MCS have demonstrated that boys, certain ethnic minorities (Indian, Pakistan/Bangladeshi and Black Caribbean/African), and children living with only one parent were less likely to provide valid accelerometer data.[55] Drop-out analyses showed that the participants included in this analysis were more likely to be male and come from a higher socioeconomic background. It has previously been shown that the presence of a missing-not-at-random mechanism that underestimates the volume of physical activity during weekend days in this cohort.[56] This mechanism may have influenced our findings. The findings of our models need to be interpreted in consideration of these limitations and our inclusion criteria, specifically our restriction to participants with at least 10 hours of wear time across 3 days. Lastly, accelerometers underestimate activity involving vertical movement (e.g. cycling) and those for which the accelerometer was not to be worn (e.g. aquatic activities or contact sports). If these behaviours are also socio-economically or ethnically patterned, this may have led to an under or overestimation of the true associations.

For peer review only - http://bmjopen.bmj.com/site/about/guidelines.xhtml
For peer review only

Our supplementary analyses support the well-established notion that VPA is more strongly associated with BMI z-score than MPA,[13,15–17] and point to a relevant effect size at a population level.[10] There are multiple reasons why the differences in VPA observed may exist. Due to unequal access and costs, the organized contexts through which children accumulate VPA have been shown to result in differences in participation between more and less advantaged subgroups of children.[24–26] These findings are however based on questionnaire-based assessments which capture sports and organized activity more accurately than other types of activity.[57] Additional factors, including parental perceptions of time commitments, and the limited variety of activities accessible, are significant factors linked to low levels of vigorous physical activity in low income families.[58] Furthermore, differences in home and family support for physical activity have been demonstrated between ethnic groups.[27] For instance, the presence of cultural and religious barriers have been found to impede participation in organized activity amongst Pakistani and Bangladeshi children, who in this analysis had the lowest levels of VPA. Our findings also demonstrate that differences in VPA between children from high and low maternal education families are more pronounced during the week. This may result from factors such as longer, inconsistent work hours within low-income jobs. These differences and differential barriers should be considered when developing interventions to increase VPA, and potentially impact obesity prevalence, in all groups. Furthermore, this finding highlights the potential of school-based and after-school sports clubs to contribute to a solution for reducing existing behavioural inequalities. Despite known differences in boys' and girls' MVPA,[59,60] also in this cohort,[21] the lack of gender interaction in this analysis reveal that both girls' and boys' participation in VPA are equally affected by socioeconomic and ethnic factors.

International physical activity guidelines for children focus on MVPA in part because increases in MVPA are hypothesised to be easier to achieve than VPA at a population level.[61] Our findings indicate that by utilizing the aggregate measure of MVPA we may be overlooking significant differences in the relative participation in MPA and VPA between population subgroups and tolerating substantial inequalities in the most important segment of physical activity for health outcomes. UK activity guidelines additionally recommend that children 'minimise the amount of time spent being sedentary for extended periods' and that 'vigorous intensity activities be incorporated at least three times a week'. Our results provide further empirical evidence to support the findings of Richards et al. (2015),[62] and their accompanying call to place more attention on the VPA component of guidelines to ensure health benefits. Further evidence suggests that childhood participation in sporting activities is vital to the development of fundamental motor skills, which strongly predict physical activity and weight status both in childhood and throughout adulthood.[63] To lay a foundation for lifelong activity participation it is critical that children, especially those from disadvantaged backgrounds, are provided with sufficient opportunities to develop fundamental motor skills. However, there is currently insufficient evidence concerning the most appropriate daily dose of VPA,

For peer review only - http://bmjopen.bmj.com/site/about/guidelines.xhtml

and how to effectively promote VPA across population subgroups. Research efforts are needed to develop effective interventions for increasing VPA.

For peer review only

CONCLUSION

We found that the amount of vigorous intensity activity accumulated was socio-economically and ethnically patterned in 7-year-old UK children, mirroring known inequalities in adiposity.[50] These findings suggest that the current central focus of physical activity guidelines, and accordingly interventions, on the promotion of aggregate measures of MVPA, may be masking behavioural differences that may have an influential role in widening inequalities in obesity between more and less advantaged subgroups. In efforts to combat rising and widening childhood obesity rates, our results suggest a need for a greater focus on the promotion of VPA in health promotion efforts, particularly for those children from more disadvantaged backgrounds.

For peer review only

For peer review only - http://bmjopen.bmj.com/site/about/guidelines.xhtml

ACKNOWLEDGEMENTS

The co-operation of the participating families is gratefully acknowledged. The fourth sweep of the MCS was funded by grants to Professor Heather Joshi, former director of the study, from the Economic and Social Research Council and a consortium of government funders. The current director is Professor Lucinda Platt. The authors acknowledge the Centre for Longitudinal Studies, Institute of Education, for the use of these data; the UK Data Service for making them available; and the MRC Centre of Epidemiology for Child Health (grant reference G0400546), Institute of Child Health, University College London, for creating the accelerometer data resource, which was funded by the Wellcome Trust (grant reference 084686/ Z/08/A). The institutions and funders acknowledged bear no responsibility for the analysis or interpretation of these data. We also would like to thank Stephen Sharp at the MRC Epidemiology Unit for his statistical insights and input on the models.

AUTHOR'S CONTRIBUTIONS

RL and EvS designed the study. JA and AA provided inputs on preliminary results and contributed to redeveloping the statistical models. RL conducted all of the analyses and drafted the manuscript. All authors contributed to the interpretation of the results and critically reviewed the manuscript. All authors read and approved the final manuscript. In addition, RL is the guarantor and responsible for the overall content.

For peer review only

References

1. Abarca-Gómez L, Abdeen ZA, Hamid ZA, et al. Worldwide trends in body-mass index, underweight, overweight, and obesity from 1975 to 2016: a pooled analysis of 2416 population-based measurement studies in 128·9 million children, adolescents, and adults. *Lancet*. 2017;0(0):1-16. doi:10.1016/S0140-6736(17)32129-3.

2. Kelly T, Yang W, Chen C-S, Reynolds K, He J. Global burden of obesity in 2005 and projections to 2030. *Int J Obes (Lond)*. 2008;32(9):1431-1437. doi:10.1038/ijo.2008.102.

3. Reilly J, Kelly J. Long-term impact of overweight and obesity in childhood and adolescence on morbidity and premature mortality in adulthood: systematic review. *Int J Obes*. 2010;35(10):891-898. doi:10.1038/ijo.2010.222.

4. UK Government Department for International Development. Agenda 2030: Delivering the Global Goals. 2017;(March 2017):29 f. https://www.gov.uk/government/publications/agenda-2030-delivering-the-global-goals.

5. Goisis A, Sacker A, Kelly Y. Why are poorer children at higher risk of obesity and overweight ? A UK cohort study. 2015:1-7. doi:10.1093/eurpub/ckv219.

6. Hargreaves DS, Marbini AD, Viner RM. Inequality trends in health and future health risk among English children and young people, 1999-2009. *Arch Dis Child*. 2013;98(11):850-855. doi:10.1136/archdischild-2012-303403.

7. Shackleton N, Hale D, Viner RM. Trends and socioeconomic disparities in preadolescent's health in the UK: Evidence from two birth cohorts 32 years apart. *J Epidemiol Community Health*. 2015;70(2):140-146. doi:10.1136/jech-2015-205603.

8. Martinson ML, Mclanahan S, Brooks-gunn J. Race/Ethnic and Nativity Disparities in Child Overweight in the United States and England. 2012;643(1):219-238. doi:10.1177/0002716212445750.Race/Ethnic.

9. Zilanawala A, Davis-Kean P, Nazroo J, Sacker A, Simonton S, Kelly Y. Race/ethnic disparities in early childhood BMI, obesity and overweight in the United Kingdom and United States. *Int J Obes*. 2015;39(3):520-529. doi:10.1038/ijo.2014.171.

10. Ekelund U. Moderate to Vigorous Physical Activity and Sedentary Time and Cardiometabolic Risk Factors in Children and Adolescents. *JAMA*. 2012;307(7):704. doi:10.1001/jama.2012.156.

11. Janssen I, Katzmarzyk PT, Boyce WF, et al. Comparison of overweight and obesity prevalence in school-aged youth from 34 countries and their relationships with physical activity and dietary patterns. *Obes Rev*. 2005;6(2):123-132. doi:10.1111/j.1467-789X.2005.00176.x.

12. Steinbeck KS. The importance of physical activity in the prevention of overweight and obesity in childhood: a review and an opinion. *Obes Rev*. 2001;2(2):117-130. doi:10.1046/j.1467-789x.2001.00033.x.

13. Steele RM, van Sluijs EM, Sharp SJ, Landsbaugh JR, Ekelund U, Griffin SJ. An investigation of patterns of children's sedentary and vigorous physical activity throughout the week. *Int J Behav Nutr Phys Act*. 2010;7:88. http://ovidsp.ovid.com/ovidweb.cgi?T=JS&PAGE=reference&D=prem&NEWS=N&AN=21143901.

14. Pavey TG, Peeters G, Bauman AE, Brown WJ. Does Vigorous Physical Activity Provide Additional Benefits beyond Those of Moderate? *Med Sci Sport Exerc*. 2013;45(10):1948-1955. doi:10.1249/MSS.0b013e3182940b91.

For peer review only

For peer review only - http://bmjopen.bmj.com/site/about/guidelines.xhtml

15. Carson V, Rinaldi RL, Torrance B, et al. Vigorous physical activity and longitudinal associations with cardiometabolic risk factors in youth. 2013;(July):16-21. doi:10.1038/ijo.2013.135.

16. Owens S, Galloway R, Gutin B. The case for vigorous physical activity in youth. *Am J Lifestyle Med*. 2017;11(2):96-115. doi:10.1177/1559827615594585.

17. Gutin B, Zenong Y, Humphries M, Barbeau P. Relations of moderate and vigorous physical activity to fitness and fatness in adolescents. *Am J Clin Nutr*. 2005;(81):746-750.

18. Griffiths LJ, Sera F, Cortina-Borja M, Law C, Ness A, Dezateux C. Objectively measured physical activity and sedentary time: Cross-sectional and prospective associations with adiposity in the millennium cohort study. *BMJ Open*. 2016;6(4). doi:10.1136/bmjopen-2015-010366.

19. Riddoch CJ, Mattocks C, Deere K, et al. Objective measurement of levels and patterns of physical activity. *Arch Dis Child*. 2007;92(11):963-969. doi:10.1136/adc.2006.112136.

20. Ruiz JR, Ortega FB, Martínez-Gómez D, et al. Objectively measured physical activity and sedentary time in European adolescents: the HELENA study. *Am J Epidemiol*. 2011;174(2):173-184. doi:10.1093/aje/kwr068.

21. Griffiths LJ, Cortina-Borja M, Sera F, et al. How active are our children? Findings from the Millennium Cohort Study. *BMJ Open*. 2013;3(8):e002893-e002893. doi:10.1136/bmjopen-2013-002893.

22. Bammann K, Gwozdz W, Pischke C, et al. The impact of familial , behavioural and psychosocial factors on the SES gradient for childhood overweight in Europe . A longitudinal study. 2016;41(1):54-60. doi:10.1038/ijo.2016.137.

23. Butte NF, Watson KB, Ridley K, et al. A Youth Compendium of Physical Activities. *Med Sci Sport Exerc*. 2017:1. doi:10.1249/MSS.0000000000001430.

24. Marques A, Ekelund U, Sardinha LB. Associations between organized sports participation and objectively measured physical activity, sedentary time and weight status in youth. *J Sci Med Sport*. 2015:2-5. doi:10.1016/j.jsams.2015.02.007.

25. Fernandes R a, Reichert FF, Monteiro HL, et al. Characteristics of family nucleus as correlates of regular participation in sports among adolescents. *Int J Public Health*. 2012;57(2):431-435. doi:10.1007/s00038-010-0207-7.

26. Hardy LL, Kelly B, Chapman K, King L, Farrell L. Parental perceptions of barriers to children's participation in organised sport in Australia. *J Paediatr Child Health*. 2010;46(4):197-203. doi:10.1111/j.1440-1754.2009.01661.x.

27. Trigwell J, Murphy RC, Cable NT, Stratton G, Watson PM. Parental views of children ' s physical activity : a qualitative study with parents from multi-ethnic backgrounds living in England. 2015:1-11. doi:10.1186/s12889-015-2351-8.

28. Corder K, Sharp SJ, Atkin AJ, et al. Age-related patterns of vigorous-intensity physical activity in youth: The International Children's Accelerometry Database. *Prev Med Reports*. 2016;4:17-22. doi:10.1016/j.pmedr.2016.05.006.

29. Hansen K, Jones E, Joshi H. *Millennium Cohort Study Fourth Survey : A User ' s Guide to Initial Findings*. London; 2010.

30. Studies C for L. *Millennium Cohort Study: A Guide to the Datasets (Eight Edition); First, Second, Third, Fourth and Fifth Surveys.* London; 2014.

For peer review only

For peer review only - http://bmjopen.bmj.com/site/about/guidelines.xhtml

31. Marco Geraci A, Rich C, Sera F, Cortina-Borja M, Griffiths LJ. *Technical Report on Accelerometry Data Processing in the Millennium Cohort Study*.; 2012. http://discovery.ucl.ac.uk/1361699/2/1361699_updated_Technical Report Accelerometry Data Processing_121106.pdf. Accessed April 4, 2016.

32. Ott A, Pate R, Trost S, Ward D, Saunders R. The Use of Uniaxial and Triaxial Accelerometers to Measure Children's "Free-Play" Physical Activity. *Pediatr Exerc Sci*. 2000;12(4):360-370.

33. Trost S, Ward D, Moorehead S, Watson P, Riner W, Burke J. Validity of the computer science and applications (CSA) activity monitor in children. *Med Sci Sport Exerc*. 1998;30(4):629-633.

34. Puyau MR, Adolph AL, Vohra FA, Butte NF. Validation and calibration of physical activity monitors in children. *Obes Res*. 2002;10(3):150-157. doi:10.1038/oby.2002.24.

35. Mattocks C, Ness A, Leary S, et al. Use of accelerometers in a large field-based study of children: protocols, design issues, and effects on precision. *J Phys Act Health*. 2008;5 Suppl 1:S98-111. http://www.ncbi.nlm.nih.gov/pubmed/18364528. Accessed April 26, 2018.

36. Shrewsbury V, Wardle J. Socioeconomic Status and Adiposity in Childhood: A Systematic Review of Cross-sectional Studies 1990–2005. *Obesity*. 2008;16(2):275-284. doi:10.1038/oby.2007.35.

37. Zhang Q, Wang Y. Socioeconomic inequality of obesity in the United States: Do gender, age, and ethnicity matter? *Soc Sci Med*. 2004;58(6):1171-1180. doi:10.1016/S0277-9536(03)00288-0.

38. Braveman PA, Cubbin C, Egerter S, Williams DR, Pamuk E. Socioeconomic disparities in health in the United States: What the patterns tell us. *Am J Public Health*. 2010;100(SUPPL. 1). doi:10.2105/AJPH.2009.166082.

39. Harding S, Teyhan A, Maynard MJ, Cruickshank JK. Ethnic differences in overweight and obesity in early adolescence in the MRC DASH study: The role of adolescent and parental lifestyle. *Int J Epidemiol*. 2008;37(1):162-172. doi:10.1093/ije/dym252.

40. Cole T, Law C, MCS Child Health Group. An ecological systems approach to examining risk factors for early childhood overweight : findings from the UK Millennium. *J Epidemiol Community Heal*. 2009;63(2):147-155. doi:10.1136/jech.2008.077917.An.

41. Pillas D, Marmot M, Naicker K, Goldblatt P, Morrison J, Pikhart H. Social inequalities in early childhood health and development: A European-wide systematic review. *Pediatr Res*. 2014;76(5):418-424. doi:10.1038/pr.2014.122.

42. Rosenberg R. *MCS4 : Guide to Derived Variables*.; 2012.

43. Statistics O for N. *Ethnic Group Statistics: A Guide for the Collection and Classification of Ethnicity Data*. London; 2003.

44. Green MJ, Popham F. Interpreting mutual adjustment for multiple indicators of socioeconomic position without committing mutual adjustment fallacies. *BMC Public Health*. 2019;19(1):1-7. doi:10.1186/s12889-018-6364-y.

45. Fairclough SJ, Boddy LM, Mackintosh KA, Valencia-Peris A, Ramirez-Rico E. Weekday and weekend sedentary time and physical activity in differentially active children. *J Sci Med Sport*. 2015;18(4):444-449. doi:10.1016/j.jsams.2014.06.005.

46. StataCorp. Chapter 20: Estimation and Postestimation commands. In: *Stata 13 Base Reference Manual*. ; 2010. https://www.stata.com/manuals13/u20.pdf.

For peer review only

47. Huber P. The behavior of maximum likelihood estimates under nonstandard conditions. *Fifth Berkeley Symp Math Stat Probab*. 1967;1:221-233. doi:citeulike-article-id:2607115.

48. Griffiths L, Rich C, Geraci M, et al. *Technical Report on the Enhancement of Millennium Cohort Study Data with Accelerometer-Derived Measures of Physical Activity and Sedentary Behaviour in Seven Year Olds*.; 2013.

49. Newton HJ, Baum CF, Beck N, et al. The Stata Journal. *Stata J*. 2010;10(3):288-308. doi:The Stata Journal.

50. Brophy S, Cooksey R, Gravenor MB, et al. Risk factors for childhood obesity at age 5: analysis of the millennium cohort study. *BMC Public Health*. 2009;9:467. doi:10.1186/1471-2458-9-467.

51. Wilkie HJ, Standage M, Gillison FB, Cumming SP, Katzmarzyk PT. Correlates of intensity-specific physical activity in children aged 9–11 years: a multilevel analysis of UK data from the International Study of Childhood Obesity, Lifestyle and the Environment. *BMJ Open*. 2018;8(2):e018373. doi:10.1136/bmjopen-2017-018373.

52. Slootmaker SM, Schuit AJ, Chinapaw MJ, Seidell JC, van Mechelen W. Disagreement in physical activity assessed by accelerometer and self-report in subgroups of age, gender, education and weight status. *Int J Behav Nutr Phys Act*. 2009;6:17. http://ovidsp.ovid.com/ovidweb.cgi?T=JS&PAGE=reference&D=prem&NEWS=N&AN=19320985.

53. Prince SA, Adamo KB, Hamel M, Hardt J, Connor Gorber S, Tremblay M. A comparison of direct versus self-report measures for assessing physical activity in adults: a systematic review. *Int J Behav Nutr Phys Act*. 2008;5(1):56. doi:10.1186/1479-5868-5-56.

54. Wardle J, Robb K, Johnson F. Assessing socioeconomic status in adolescents: the validity of a home affluence scale. 2002:595-599.

55. Rich C, Cortina-Borja M, Dezateux C, et al. Predictors of non-response in a UK-wide cohort study of children's accelerometer-determined physical activity using postal methods. *BMJ Open*. 2013;3(3). doi:10.1136/bmjopen-2012-002290.

56. Geraci M, Farcomeni A. Probabilistic principal component analysis to identify profiles of physical activity behaviours in the presence of non-ignorable missing data. *J R Stat Soc Ser C Appl Stat*. 2016;65(1):51-75. doi:10.1111/rssc.12105.

57. Corder K, Sluijs EMF Van, Wright A, Whincup P, Wareham NJ, Ekelund U. Is it possible to assess free-living physical activity and energy expenditure in young people by self-report ? 1 – 3. 2009:862-870. doi:10.3945/ajcn.2008.26739.862.

58. Brophy S, Cooksey R, Lyons RA, Thomas NE, Rodgers SE, Gravenor MB. Parental factors associated with walking to school and participation in organised activities at age 5: analysis of the Millennium Cohort Study. *BMC Public Health*. 2011;11(1):14. doi:10.1186/1471-2458-11-14.

59. Hallal PC, Andersen LB, Bull FC, et al. Global physical activity levels: Surveillance progress, pitfalls, and prospects. *Lancet*. 2012;380(9838):247-257. doi:10.1016/S0140-6736(12)60646-1.

60. Trost SG, Pate RR, Sallis JF, et al. *Age and Gender Differences in Objectively Measured Physical Activity in Youth*. Vol 34.; 2002. http://www.acsm-msse.org. Accessed October 25, 2018.

61. Tarp J, Child A, White T, et al. Physical activity intensity, bout-duration, and cardiometabolic

For peer review only

For peer review only - http://bmjopen.bmj.com/site/about/guidelines.xhtml

risk markers in children and adolescents. *Int J Obes*. 2018:1-12. doi:10.1038/s41366-018-0152-8.

62. Richards J, Doherty A, Foster C. Is the Current Focus of the Global Physical Activity Recommendations for Youth Appropriate in All Settings ? and Cardiorespiratory Fitness Patterns for Current Global Physical Activity. *J Phys Act Heal*. 2015;12:901-903.

63. Lubans DR, Morgan PJ, Cliff DP, Barnett LM, Okely AD. Fundamental Movement Skills in Children and Adolescents Review of Associated Health Benefits. *Sport Med*. 2010;40(12):1019-1035.

For peer review only

Figure Legends

Figure 1.

Title: Proportion of VPA in daily MVPA (mins), by level of activity with participants grouped by low, middle and high socioeconomic status

Note: Tertiles of household equivalised income is used as indicator of socioeconomic status

For peer review only

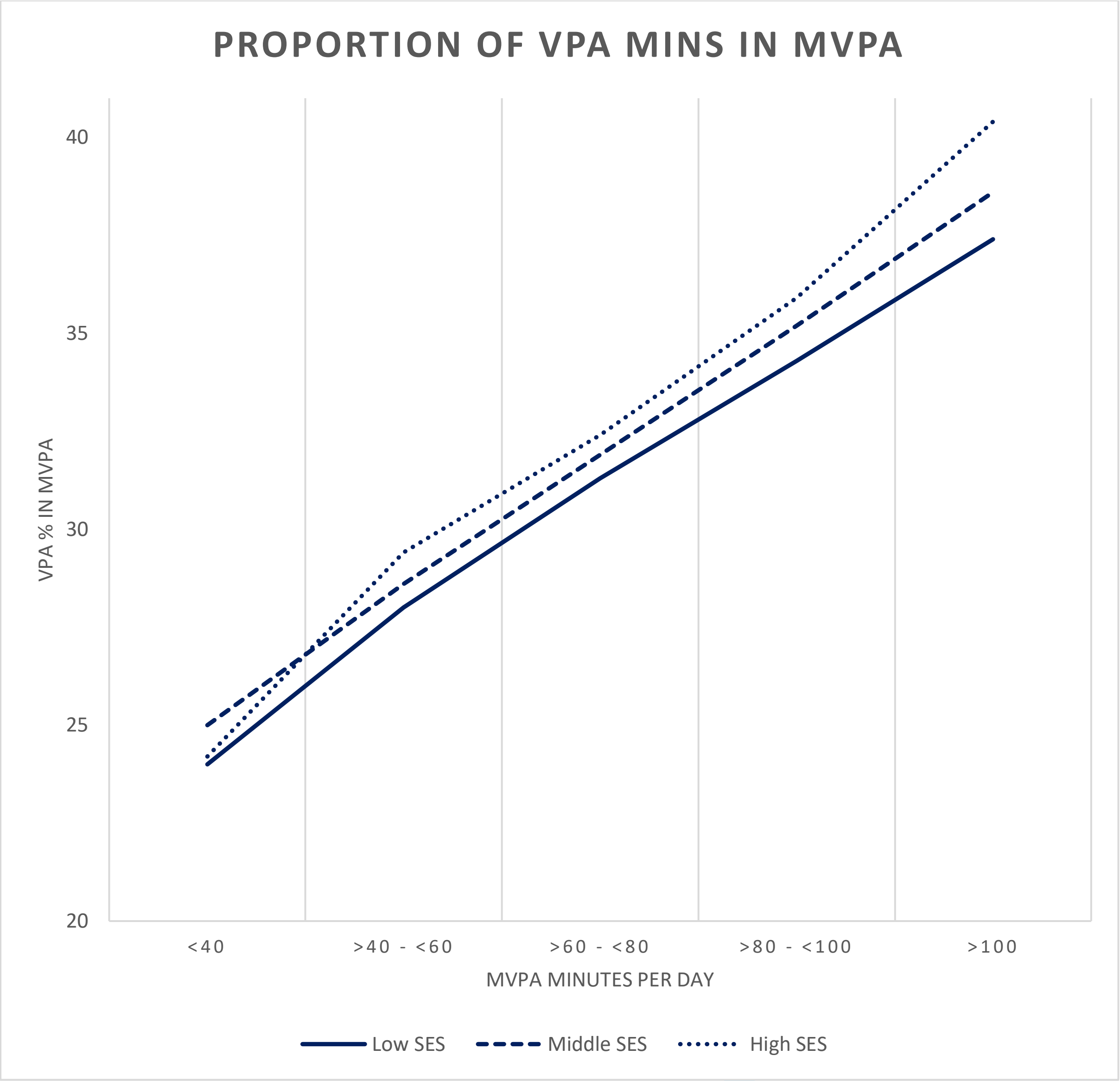
PROPORTION OF VPA MINS IN MVPA
40
35
30
25
20
VPA % IN MVPA
<40
>40 - <60
>60 - <80
>80 - <100
>100
MVPA MINUTES PER DAY
Low SES
Middle SES
High SES

Supplementary Files: Socio-economic and ethnic differences in children's vigorous intensity physical activity: a cross-sectional analysis of the UK Millennium Cohort Study

Contents:

Supplementary File 1: Full multivariable linear regression models for mean minutes of VPA and MPA overall, on weekdays and on weekend days, by a) ethnic subgroups b) maternal education and c) household equivalised income (n=5172) within the Millennium Cohort Accelerometer Study, 4th follow-up (2008-09)

Supplementary File 2: Multivariable linear regression models for mean minutes of VPA with additional adjustments for BMI z-score overall, on weekdays and on weekend days, by ethnic subgroups maternal education and household equivalised income (n=5149) within the Millennium Cohort Accelerometer Study, 4th follow-up (2008-09)

Supplementary File 3: Multivariable linear regression models for BMI z-score by minutes of daily VPA and MPA (n=5149) within the Millennium Cohort Accelerometer Study, 4th follow-up (2008-09)

For peer review only

Supplementary File 1a) Multivariable linear regression models for mean minutes of VPA and MPA overall, weekdays and weekend days, by ethnic subgroups (n=5172) within the Millennium Cohort Accelerometer Study, 4th follow-up (2008-09)

**By ethnic group**			note: .01 - ***; .05 - **; .1 - *;		
**VPA (mins/day)**			**MPA (mins/day)**		
**All valid days**					
**ref cat: White**	**coef**	**se**	**ref cat: White**	**coef**	**se**
Mixed	1.468*	0.782	Mixed	-2.223**	1.036
Indian	0.738	0.984	Indian	-3.938***	1.168
Pakistani & Bangladeshi	-3.344***	0.673	Pakistani & Bangladeshi	2.089**	0.900
Black or Black British	-1.675	1.168	Black or Black British	3.009***	0.988
Other ethnic group	-2.267**	0.966	Other ethnic group	1.087	1.728
MPA (mins/day)	0.584***	0.018	VPA (mins/day)	0.814***	0.020
Weartime (mins/day)	0.000	0.003	Weartime (mins/day)	0.014***	0.003
Age (yrs)	1.079**	0.535	Age (yrs)	-1.876***	0.657
Sex of participant	0.700**	0.292	Sex of participant	-5.064***	0.320
Season of measurement	0.042	0.165	Season of measurement	-1.727***	0.177
_cons	-14.150***	4.293	_cons	41.945***	5.295
r2	0.515		r2	0.565	
Number of observations	5,172		Number of observations	5,172	
**Weekdays**					
**ref cat: White**	**coef**	**se**	**ref cat: White**	**coef**	**se**
Mixed	1.569*	0.871	Mixed	-2.001*	1.138
Indian	0.578	0.923	Indian	-3.463***	1.106
Pakistani & Bangladeshi	-3.451***	0.743	Pakistani & Bangladeshi	1.998**	0.965
Black or Black British	-2.076*	1.223	Black or Black British	3.098***	1.167
Other ethnic group	-3.069***	1.145	Other ethnic group	1.888	1.891
Weekday MPA (mins/day)	0.584***	0.022	Weekday VPA (mins/day)	0.789***	0.021
Weekday weartime (mins/day)	-0.000	0.003	Weekday weartime (mins/day)	0.015***	0.003
Age (yrs)	0.951*	0.576	Age (yrs)	-1.331*	0.703
Sex of participant	0.739**	0.311	Sex of participant	-5.066***	0.332
Season of measurement	0.026	0.171	Season of measurement	-1.693***	0.184
_cons	-12.724***	4.553	_cons	37.062***	5.484
r2	0.497		r2	0.543	
Number of observations	5,172		Number of observations	5,172	
**Weekend days**					
**ref cat: White**	**coef**	**se**	**ref cat: White**	**coef**	**se**
Mixed	0.434	0.944	Mixed	-2.131	1.484
Indian	0.757	1.499	Indian	-4.463**	1.785
Pakistani & Bangladeshi	-3.001***	0.692	Pakistani & Bangladeshi	2.024*	1.198
Black or Black British	-0.035	1.736	Black or Black British	1.766	1.390
Other ethnic group	-0.141	1.091	Other ethnic group	-1.339	2.059
Weekend MPA (mins/day)	0.540***	0.016	Weekend VPA (mins/day)	0.956***	0.023
Weekend weartime (mins/day)	-0.002	0.002	Weekend weartime (mins/day)	0.017***	0.003
Age (yrs)	1.389**	0.689	Age (yrs)	-3.486***	0.918
Sex of participant	0.103	0.331	Sex of participant	-4.643***	0.466
Season of measurement	-0.205	0.217	Season of measurement	-1.483***	0.270
_cons	-12.259**	5.199	_cons	48.524***	7.067
r2	0.546		r2	0.572	
Number of observations	5,172		Number of observations	5,172	

For peer review only

Supplementary File 1b) Multivariable linear regression models for mean minutes of VPA and MPA overall, weekdays and weekend days, by level of maternal education (n=5172) within the Millennium Cohort Accelerometer Study, 4th follow-up (2008-09)

**By level of maternal education**			note: .01 - ***; .05 - **; .1 - *;		
**VPA (mins/day)**			**MPA (mins/day)**		
**All valid days**					
**ref cat: No qualifications**	**coef**	**se**	**ref cat: No qualifications**	**coef**	**se**
Low	1.314**	0.559	Low	-0.950	0.836
Medium	1.653***	0.572	Medium	-1.483*	0.851
High	1.812***	0.532	High	-1.689**	0.815
Higher	2.957***	0.769	Higher	-2.738***	0.925
Overseas qual.	2.276*	1.314	Overseas qual.	-1.957	1.334
None of these	-0.448	0.674	None of these	1.978*	1.035
MPA (mins/day)	0.587***	0.018	VPA (mins/day)	0.815***	0.019
Weartime (mins/day)	-0.002	0.003	Weartime (mins/day)	0.014***	0.003
Age (yrs)	1.044*	0.543	Age (yrs)	-1.901***	0.656
Sex of participant	0.681**	0.297	Sex of participant	-5.043***	0.325
Season of measurement	0.081	0.164	Season of measurement	-1.736***	0.179
_cons	-14.404***	4.418	_cons	42.866***	5.471
r2	0.515		r2	0.567	
Number of observations	5,172		Number of observations	5,172	
**Weekdays**					
**ref cat: No qualifications**	**coef**	**se**	**ref cat: No qualifications**	**coef**	**se**
Low	1.314**	0.616	Low	-0.944	0.893
Medium	1.717***	0.628	Medium	-1.384	0.907
High	1.880***	0.585	High	-1.732**	0.869
Higher	3.045***	0.843	Higher	-2.708***	0.962
Overseas qual.	2.123	1.314	Overseas qual.	-2.293	1.407
None of these	-0.597	0.727	None of these	1.878*	1.116
Weekday MPA (mins/day)	0.588***	0.021	Weekday VPA (mins/day)	0.790***	0.021
Weekday weartime (mins/day)	-0.002	0.003	Weekday weartime (mins/day)	0.015***	0.003
Age (yrs)	0.910	0.584	Age (yrs)	-1.343*	0.697
Sex of participant	0.713**	0.316	Sex of participant	-5.038***	0.336
Season of measurement	0.067	0.170	Season of measurement	-1.703***	0.185
_cons	-12.864***	4.666	_cons	37.919***	5.647
r2	0.496		r2	0.545	
Number of observations	5,172		Number of observations	5,172	
**Weekend days**					
**ref cat: No qualifications**	**coef**	**se**	**ref cat: No qualifications**	**coef**	**se**
Low	1.335*	0.711	Low	-1.317	1.055
Medium	1.366*	0.736	Medium	-1.894*	1.112
High	1.627**	0.668	High	-1.711*	1.016
Higher	2.796***	0.884	Higher	-3.556***	1.194
Overseas qual.	2.694	1.714	Overseas qual.	-1.735	1.953
None of these	-0.065	0.868	None of these	2.348*	1.354
Weekend MPA (mins/day)	0.543***	0.016	Weekend VPA (mins/day)	0.955***	0.023
Weekend weartime (mins/day)	-0.002	0.002	Weekend weartime (mins/day)	0.017***	0.003
Age (yrs)	1.376**	0.698	Age (yrs)	-3.554***	0.927
Sex of participant	0.105	0.333	Sex of participant	-4.654***	0.467
Season of measurement	-0.171	0.219	Season of measurement	-1.495***	0.271
_cons	-13.489**	5.374	_cons	50.213***	7.259
r2	0.546		r2	0.575	
Number of observations	5,172		Number of observations	5,172	

For peer review only

Supplementary File 1c) Multivariable linear regression models for mean minutes of VPA and MPA overall, weekdays and weekend days, by annual household equivalised income (n=5172) within the Millennium Cohort Accelerometer Study, 4th follow-up (2008-09)

**By household equivalised income**			note: .01 - ***; .05 - **; .1 - *;		
**VPA (mins/day)**			**MPA (mins/day)**		
**All valid days**					
	**coef**	**se**		**coef**	**se**
Annual income/10000	0.579***	0.131	Annual income/10000	-0.976***	0.141
MPA (mins/day)	0.587***	0.018	VPA (mins/day)	0.812***	0.019
Weartime (mins/day)	-0.001	0.003	Weartime (mins/day)	0.014***	0.003
Age (yrs)	0.998*	0.540	Age (yrs)	-1.828***	0.654
Sex of participant	0.719**	0.299	Sex of participant	-5.109***	0.325
Season of measurement	0.103	0.166	Season of measurement	-1.772***	0.178
_cons	-14.095***	4.396	_cons	43.738***	5.339
r2	0.513		r2	0.566	
Number of observations	5,172		Number of observations	5,172	
**Weekdays**					
	**coef**	**se**		**coef**	**se**
Annual income/10000	0.613***	0.136	Annual income/10000	-0.990***	0.145
Weekday MPA (mins/day)	0.588***	0.022	Weekday VPA (mins/day)	0.786***	0.021
Weekday weartime (mins/day)	-0.002	0.003	Weekday weartime (mins/day)	0.015***	0.003
Age (yrs)	0.857	0.581	Age (yrs)	-1.272*	0.696
Sex of participant	0.753**	0.320	Sex of participant	-5.107***	0.336
Season of measurement	0.092	0.172	Season of measurement	-1.741***	0.184
_cons	-12.484***	4.629	_cons	38.716***	5.474
r2	0.494		r2	0.545	
Number of observations	5,172		Number of observations	5,172	
**Weekend days**					
	**coef**	**se**		**coef**	**se**
Annual income/10000	0.500***	0.162	Annual income/10000	-0.915***	0.210
Weekend MPA (mins/day)	0.542***	0.016	Weekend VPA (mins/day)	0.957***	0.023
Weekend weartime (mins/day)	-0.002	0.002	Weekend weartime (mins/day)	0.016***	0.003
Age (yrs)	1.346*	0.695	Age (yrs)	-3.477***	0.922
Sex of participant	0.140	0.334	Sex of participant	-4.701***	0.469
Season of measurement	-0.153	0.220	Season of measurement	-1.520***	0.272
_cons	-13.159**	5.327	_cons	50.629***	7.161
r2	0.545		r2	0.572	
Number of observations	5,172		Number of observations	5,172	

For peer review only

Supplementary file 2

Multivariable linear regression models for mean minutes of VPA with additional adjustments for BMI z-score overall, on weekdays and on weekend days, by ethnic subgroups maternal education and household equivalised income (n=5149) within the Millennium Cohort Accelerometer Study, 4th follow-up (2008-09)

Ethnicity						
	All valid days		Weekdays		Weekends	
	coef	se	coef	se	coef	se
Mixed	1.400*	0.754	1.503*	0.831	0.364	0.961
Indian	0.484	0.989	0.330	0.932	0.522	1.512
Pakistani & Bangladeshi	-3.616***	0.626	-3.735***	0.693	-3.240***	0.668
Black or Black British	-0.769	1.072	-1.124	1.139	0.678	1.680
Other ethnic group	-2.668***	0.981	-3.508***	1.161	-0.421	1.097
MPA (mins/day)	0.579***	0.018	0.581***	0.021	0.536***	0.015
Weartime (mins/day)	-0.000	0.003	-0.000	0.003	-0.002	0.002
Age (yrs)	1.069**	0.530	0.947*	0.571	1.374**	0.686
Sex of participant	0.686**	0.290	0.733**	0.309	0.099	0.330
Season of measurement	-0.007	0.162	-0.006	0.169	-0.295	0.212
BMI zscore	-0.882***	0.126	-0.932***	0.129	-0.702***	0.167
_cons	-13.169***	4.221	-11.801***	4.495	-11.436**	5.144
r2	0.523		1		1	
Number of observations	5,149		5,149		5,149	

Maternal education						
	All valid days		Weekdays		Weekends	
	coef	se	coef	se	coef	se
Low	1.385**	0.558	1.413**	0.616	1.332*	0.706
Medium	1.806***	0.572	1.882***	0.629	1.508**	0.732
High	1.841***	0.532	1.929***	0.586	1.612**	0.666
Higher	2.990***	0.765	3.093***	0.839	2.815***	0.880
Overseas qual.	2.278*	1.367	2.139	1.373	2.675	1.747
None of these	-0.452	0.668	-0.587	0.720	-0.101	0.863
MPA (mins/day)	0.584***	0.018	0.586***	0.021	0.540***	0.016
Weartime (mins/day)	-0.002	0.003	-0.002	0.003	-0.002	0.002
Age (yrs)	1.037*	0.536	0.908	0.577	1.363*	0.695
Sex of participant	0.669**	0.294	0.707**	0.313	0.102	0.332
Season of measurement	0.032	0.161	0.035	0.168	-0.260	0.213
BMI zscore	-0.876***	0.136	-0.933***	0.140	-0.668***	0.171
_cons	-13.655***	4.342	-12.143***	4.602	-12.804**	5.323
r2	0.523		0.504		0.549	
Number of observations	5,149		5,149		5,149	

Household equivalised income						
	All valid days		Weekdays		Weekends	
	coef	se	coef	se	coef	se
Annual income/10000	0.579***	0.131	0.613***	0.136	0.500***	0.162
MPA (mins/day)	0.587***	0.018	0.588***	0.022	0.542***	0.016
Weartime (mins/day)	-0.001	0.003	-0.002	0.003	-0.002	0.002
Age (yrs)	0.998*	0.540	0.857	0.581	1.346*	0.695
Sex of participant	0.719**	0.299	0.753**	0.320	0.140	0.334
Season of measurement	0.103	0.166	0.092	0.172	-0.153	0.220
_cons	-14.095***	4.396	-12.484***	4.629	-13.159**	5.327
R2	0.513		0.494		0.545	
Number of observations	5,172		5,172		5,172	

For peer review only

Supplementary file 3

Multivariable linear regression models for BMI z-score by minutes of daily VPA and MPA (n=5149) within the Millennium Cohort Accelerometer Study, 4th follow-up (2008-09), adjusted for age, sex and wear time

BMI z-score	β-coeff	[95% Conf	Interval]
Mean Minutes MPA/day	-0.002	-0.006	-0.001
Mean Minutes VPA/day	-0.012	-0.017	-0.007

For peer review only

STROBE Statement—Checklist of items that should be included in reports of ***cross-sectional studies***

	Item No	Recommendation	Page No
**Title and abstract**	1	(*a*) Indicate the study's design with a commonly used term in the title or the abstract	1
		(*b*) Provide in the abstract an informative and balanced summary of what was done and what was found	3
**Introduction**			
Background/rationale	2	Explain the scientific background and rationale for the investigation being reported	5
Objectives	3	State specific objectives, including any prespecified hypotheses	5-6
**Methods**			
Study design	4	Present key elements of study design early in the paper	7-8
Setting	5	Describe the setting, locations, and relevant dates, including periods of recruitment, exposure, follow-up, and data collection	7
Participants	6	(*a*) Give the eligibility criteria, and the sources and methods of selection of participants	7
Variables	7	Clearly define all outcomes, exposures, predictors, potential confounders, and effect modifiers. Give diagnostic criteria, if applicable	7-8
Data sources/ measurement	8*	For each variable of interest, give sources of data and details of methods of assessment (measurement). Describe comparability of assessment methods if there is more than one group	7-8
Bias	9	Describe any efforts to address potential sources of bias	9
Study size	10	Explain how the study size was arrived at	7
Quantitative variables	11	Explain how quantitative variables were handled in the analyses. If applicable, describe which groupings were chosen and why	8
Statistical methods	12	(*a*) Describe all statistical methods, including those used to control for confounding	8
		(*b*) Describe any methods used to examine subgroups and interactions	8
		(*c*) Explain how missing data were addressed	8
		(*d*) If applicable, describe analytical methods taking account of sampling strategy	8
		(*e*) Describe any sensitivity analyses	8
**Results**			
Participants	13*	(a) Report numbers of individuals at each stage of study—eg numbers potentially eligible, examined for eligibility, confirmed eligible, included in the study, completing follow-up, and analysed	9
		(b) Give reasons for non-participation at each stage	9
		(c) Consider use of a flow diagram	-
Descriptive data	14*	(a) Give characteristics of study participants (eg demographic, clinical, social) and information on exposures and potential confounders	10
		(b) Indicate number of participants with missing data for each variable of interest	9
Outcome data	15*	Report numbers of outcome events or summary measures	11
Main results	16	(*a*) Give unadjusted estimates and, if applicable, confounder-adjusted estimates and their precision (eg, 95% confidence interval). Make clear which confounders were adjusted for and why they were included	11

For peer review only - http://bmjopen.bmj.com/site/about/guidelines.xhtml

		(*b*) Report category boundaries when continuous variables were categorized	11
		(*c*) If relevant, consider translating estimates of relative risk into absolute risk for a meaningful time period	-
Other analyses	17	Report other analyses done—eg analyses of subgroups and interactions, and sensitivity analyses	9
**Discussion**			
Key results	18	Summarise key results with reference to study objectives	12
Limitations	19	Discuss limitations of the study, taking into account sources of potential bias or imprecision. Discuss both direction and magnitude of any potential bias	12
Interpretation	20	Give a cautious overall interpretation of results considering objectives, limitations, multiplicity of analyses, results from similar studies, and other relevant evidence	13
Generalisability	21	Discuss the generalisability (external validity) of the study results	13
**Other information**			
Funding	22	Give the source of funding and the role of the funders for the present study and, if applicable, for the original study on which the present article is based	2

*Give information separately for exposed and unexposed groups.

Note: An Explanation and Elaboration article discusses each checklist item and gives methodological background and published examples of transparent reporting. The STROBE checklist is best used in conjunction with this article (freely available on the Web sites of PLoS Medicine at http://www.plosmedicine.org/, Annals of Internal Medicine at http://www.annals.org/, and Epidemiology at http://www.epidem.com/). Information on the STROBE Initiative is available at www.strobe-statement.org.

For peer review only
